# Using SRM-MS to quantify nuclear protein abundance differences between adipose tissue depots of insulin-resistant mice[S]

**DOI:** 10.1194/jlr.D056317

**Published:** 2015-05

**Authors:** Asuka Ota, Kyle M. Kovary, Olivia H. Wu, Robert Ahrends, Wen-Jun Shen, Maria J. Costa, Brian J. Feldman, Fredric B. Kraemer, Mary N. Teruel

**Affiliations:** * Departments of Chemical and Systems Biology, Stanford University, Stanford, CA; † Medicine/Division of Endocrinology, Stanford University, Stanford, CA; ** Pediatrics/Division of Endocrinology, Stanford University, Stanford, CA; § Veterans Administration Palo Alto Health Care System, Palo Alto, CA

**Keywords:** adipocytes, peroxisome proliferator-activated receptors, obesity, insulin resistance, quantitative proteomics, selected reaction monitoring mass spectrometry

## Abstract

Insulin resistance (IR) underlies metabolic disease. Visceral, but not subcutaneous, white adipose tissue (WAT) has been linked to the development of IR, potentially due to differences in regulatory protein abundance. Here we investigate how protein levels are changed in IR in different WAT depots by developing a targeted proteomics approach to quantitatively compare the abundance of 42 nuclear proteins in subcutaneous and visceral WAT from a commonly used insulin-resistant mouse model, Lepr(db/db), and from C57BL/6J control mice. The most differentially expressed proteins were important in adipogenesis, as confirmed by siRNA-mediated depletion experiments, suggesting a defect in adipogenesis in visceral, but not subcutaneous, insulin-resistant WAT. Furthermore, differentiation of visceral, but not subcutaneous, insulin-resistant stromal vascular cells (SVCs) was impaired. In an in vitro approach to understand the cause of this impaired differentiation, we compared insulin-resistant visceral SVCs to preadipocyte cell culture models made insulin resistant by different stimuli. The insulin-resistant visceral SVC protein abundance profile correlated most with preadipocyte cell culture cells treated with both palmitate and TNFα. Together, our study introduces a method to simultaneously measure and quantitatively compare nuclear protein expression patterns in primary adipose tissue and adipocyte cell cultures, which we show can reveal relationships between differentiation and disease states of different adipocyte tissue types.

The prevalence of metabolic syndrome continues to increase at an alarming rate worldwide. Insulin resistance (IR) is highly associated with central obesity, and, by significantly increasing the risk of developing type 2 diabetes and cardiovascular disease, poses a great problem to human health. However, current therapeutics for IR are primarily limited to weight loss and bariatric surgery and are not effective for all patients. To provide alternative therapies, it is essential to understand the molecular mechanisms underlying IR. Adipocytes play a central role in regulating whole body metabolism, mainly by secreting key hormones and cytokines, as well as lipids. The functions of adipocytes are affected in IR, leading to increased inflammation, dysregulation of lipid metabolism, and changes in the adipocyte state (1, 2).

White adipose tissues (WATs) are frequently separated into two different types: subcutaneous and visceral. Recent studies show that visceral WAT is strongly associated with IR, and its tissue mass is an independent risk factor for many metabolic diseases (3–5). In contrast, subcutaneous WAT is important for increasing insulin sensitivity and has been shown to be metabolically protective (6, 7). Thus, understanding the functional and developmental differences between visceral and subcutaneous adipocytes in insulin-resistant animals may provide insight into restoring systemic insulin sensitivity by converting visceral adipocytes to subcutaneous-like adipocytes.

We hypothesized that functional distinctions between different types of adipocytes could be discerned by profiling the expression levels of a group of nuclear proteins that are important for differentiation or gene regulation. Here, we isolated visceral (epididymal) and subcutaneous (inguinal) adipocytes from insulin-resistant Lepr(db/db) mice and control C57BL/6J mice and quantified expression levels of nuclear proteins in these primary adipocytes using our novel approach of selected reaction monitoring MS (SRM-MS). This approach provides quantification of up to hundreds of proteins simultaneously and requires a fraction of the protein that would be needed for the equivalent protein quantification by Western blot. We found that many proteins that were differentially expressed in these adipocytes affected adipogenesis. We then compared the protein abundance profiles from primary stromal vascular cells (SVCs), which are enriched for preadipocytes, to different cell culture models of IR generated by treating OP9 cells with IR-inducing compounds. The results showed that the nuclear protein profile of db/db visceral SVCs is most similar to that of OP9 preadipocytes treated with both TNFα and palmitate (PA). Together our study introduces a targeted proteomics approach that can be applied to profile and correlate different types of insulin-resistant adipocytes and preadipocytes, both from primary adipose tissue depots and from cell culture. Understanding the molecular mechanisms underlying adipocyte dysfunction in insulin-resistant animals is important to enable the development of more targeted therapeutics to treat IR.

## MATERIALS AND METHODS

### Glucose and insulin tolerance tests

Nine-week-old C57BL/6J (control) and db/db male mice were purchased from Jackson Laboratory and housed in the animal facility at Stanford University or at the Veterans Administration Palo Alto Health Care System until they were 10 weeks old. The db/db and control mice were in the same genetic background. All animal studies were approved by the Institutional Animal Care and Use Committees at Stanford and Veterans Administration Palo Alto Health Care System. For insulin tolerance tests (ITTs), mice were fasted overnight. At 9:00 AM of the following day, the mice were weighed, and their baseline blood glucose levels were measured from a tail vein using a glucose meter (Bayer Contour). Insulin was injected intraperitoneally at the concentration of 0.75 U per kilogram of body weight, and the blood glucose level was measured at indicated times. Mice were fed after the ITT, and a glucose tolerance test (GTT) was performed 3 days later. Glucose levels prior to and after intraperitoneal injection of 1 g of glucose per kilogram of body weight were measured at the indicated times.

### Isolation of primary adipocytes and SVCs

Adipocytes and SVCs were isolated from visceral and subcutaneous WAT from 10-week-old db/db and C57BL/6J mice. To obtain enough nuclear proteins for each SRM-MS sample, tissues were pooled from either six C57BL/6J mice or two db/db mice. WAT was excised from the different fat depots in the mice, finely minced, and digested with collagenase type I (1 mg collagenase/ml of HEPES buffer; 3 ml/g of tissue) at 37°C for 35–40 min, filtered to exclude large debris, and singly isolated cells were collected. The cells were separated into the adipocyte (supernatant) and SVC fractions (pellet) by spinning cells at 150–200 relative centrifugal force (RCF) for 3 min. Each fraction was rinsed three times in PBS with 3% BSA. The SVC fraction, which contained preadipocytes, was plated in culture medium (DMEM with 10% calf serum, 100 U/ml penicillin/streptomycin, and 1 μg/ml of amphotericin B) for 4 h and then washed with culture medium to remove cell debris and nonadherent cells, thus enriching for preadipocytes which adhere well to the culture surface. Cells were further grown in culture for one to two passages before performing experiments. Mature adipocytes were resuspended in buffer A [10 mM HEPES (pH 7.9), 1.5 MgCl_2_, 10 ml KCl, 1 mM PMSF, and protease inhibitor (Roche)], and were used immediately to isolate nuclear proteins.

### Cell culture

OP9 cells were grown following previously published protocols (8, 9). Briefly, cells were grown in MEMα with L-glutamine, 20% FBS, and 100 U/ml penicillin/streptomycin. To determine the effect of TNFα on insulin sensitivity, OP9 preadipocytes were treated with 5 ng/ml TNFα (Sigma-Aldrich T7539) or water (control) for 24 h. To determine the effect of PA on insulin sensitivity, 250 mM PA (Sigma-Aldrich P9767) was first dissolved in 95% ethanol and mixed with 10% fatty acid-free BSA (Sigma-Aldrich A8806) in MEMα medium at a final concentration of 5 mM. The 5 mM mixture was then incubated at 37°C for 1 h to form PA-BSA complexes. Then the 5 mM mixture of PA-BSA complexes was diluted 10-fold in MEMα and applied to the cells for 24 h. For combined TNFα and PA treatment, cells were treated with both 5 ng/ml TNFα and 0.5 mM PA for 24 h. For the PA or TNFα+PA treatments, treatment with 3.8% BSA-ethanol complexes was used as the control.

To determine the effect of TNFα or PA on adipogenesis, OP9 cells were plated at 100% confluency and treated by adding TNFα, PA, TNFα+ PA, or the appropriate control to MDI medium. MDI medium consists of MEMα containing 10% FBS, 100 U/ml penicillin/streptomycin, 0.5 mM isobutyl-methylxanthine, 1 μM dexamethasone, and 175 μM insulin. After 48 h, the medium was replaced with insulin medium (MEMα containing 10% FBS, 100 U/ml penicillin/streptomycin, and 175 μM insulin) to which TNFα, PA, TNFα+PA, or the appropriate control had been added. Cells were fully differentiated 4 days after the addition of MDI medium. To measure phospho-AKT (pAKT) levels, cells were starved in 0.5% FBS in MEMα for 4 h, stimulated with 1 μM insulin for 10 min, and then fixed in preparation for immunocytochemistry analysis.

### Sample preparation for SRM-MS

To isolate nuclei from OP9 cells, cells were rinsed in PBS and dounced through 30 gauge needles in buffer A to break open the cells. The cell lysate was then centrifuged at 2,500 RCF for 10 min at 4°C to pellet the nuclei. To isolate nuclei from primary adipocytes, cells isolated and resuspended in buffer A were broken open using a glass-glass dounce homogenizer and centrifuged at 40,000 RCF for 45 min at 4°C over a 0.5–1.25 M sucrose gradient. Isolated nuclei were rinsed twice and pelleted in buffer A by centrifuging at 2,500 RCF for 10 min at 4°C. Nuclear pellets were resuspended in a high salt buffer consisting of 20 mM HEPES (pH 7.9), 25% v/v glycerol, 450 mM KCl, 1.5 mM MgCl_2_, 0.2 mM EDTA, 1 mM DTT, 1 mM PMSF, and EDTA-free complete protease inhibitor cocktail (Roche) to extract the soluble nuclear protein fraction. Samples were placed on ice for 15 min followed by gentle shaking every 5 min for an additional 15 min before cen­trifuging at 16,100 *g* for 10 min to pellet out the histones and DNA. Proteins were precipitated by adding at least 5 vol of ice-cold acetone and placed at −20°C overnight. Precipitated proteins were centrifuged at 18,000 *g* for 20 min at 4°C. Protein pellets were resuspended in 8 M urea to denature the proteins, and were then diluted to 2 M urea with 50 mM ammonium bicarbonate. Protein concentrations were measured using the BCA protein assay (Thermo Scientific). Disulfide bonds were reduced by treating proteins with 10 mM Tris (2-carboxyethyl)phosphine hydrochloride for 30 min at 37°C. The free thiol groups on the reduced cysteines were alkylated with 15 mM iodoacetamide at room temperature for 30 min. The samples were then diluted to 1 M urea with 50 mM ammonium bicarbonate before adding synthetic heavy peptides (JPT Peptides, Berlin, Germany) for each target protein at a ratio of 130 femtomoles per 10 μg of total protein. Sequencing-grade modified trypsin (Promega) was added at a ratio of 1 μg per 100 μg of protein, and the proteins were digested overnight at 37°C. The peptides were acidified to pH 2–3 with formic acid, desalted on C18 Sep-Pak cartridges (Waters), and evaporated on a lyophilizer. The peptides were resolubilized in 2% acetonitrile with 0.1% formic acid at a concentration of 1 μg/μl.

The purity of the nuclear fraction was verified by using SRM-MS to monitor control cytosolic and nuclear proteins (supplementary Fig. 1).

### SRM-based peptide quantification

As detailed in (10), all peptides used in this study were carefully selected, verified for robustness and specificity, and successfully applied in a previous study to quantify proteins in OP9 cells, an adipocyte cell culture model. Following the procedures described in (10), to quantify proteins, the peptides were separated on a Proxeon EASY-nLC Nano-HPLC system (Proxeon, Odense, Denmark), and were introduced into a TSQ Vantage triple quadrupole mass spectrometer (Thermo Fisher Scientific, Bremen, Germany) via a Proxeon nanospray ionization source. Peptides representing 2 μg of total protein were loaded onto a 25 mm × 0.1 mm C18 trapping column (MICHROM C18, 5 μm, 120 Å) and a 200 mm × 0.075 mm diameter reverse-phase C18 capillary column (Maisch C18, 3 μm, 120 Å) and were subjected to a linear gradient from 0 to 45% acetonitrile over 70 min at a flow rate of 300 nl/min.

To quantify our proteins of interest, we identified prototypic peptides and ordered corresponding synthetic heavy labeled peptides. The heavy peptides (Spiketides) used as internal standards were obtained from JPT Peptides and were isotopically labeled, ensuring that they coeluted exactly with the endogenous peptides and the peak areas could be directly ratioed. To make the heavy peptides, the C-terminal amino acid lysine or arginine residue was substituted with a heavy version containing 13C and 15N atoms which resulted in a mass shift of +8 Da for lysine or +10 Da for arginine. Heavy peptides containing cysteine residues were synthesized with alkylated cysteines. Because the Spiketides used as heavy standards were not absolutely quantified, all protein measurements represent relative, not absolute, abundance.

As described in detail in (10), the heavy peptides served to both validate and quantify endogenous peptide transitions. Peptide validation was defined by the coelution of both the synthetic (heavy) and endogenous (light) peptides, as well as matching fragmentation patterns. Also, many peptides were further validated by carrying out siRNA-mediated depletion experiments. Different peptides from the same protein showed similar changes in the same direction. However, as we also found in the current study, the raw values for different peptides from the same protein differed by several fold in some cases. Thus, as in (10), we selected a single representative peptide from each protein and used the same peptide for all the samples to do the relative quantification between samples.

The heavy and light peptide transitions were measured using scheduled SRM-MS. Typically three transitions were measured per peptide. All peptides and transitions used in this study are listed in supplementary Table 1. SRM-MS traces were analyzed using Skyline version 2.5 software (MacCoss Lab, University of Washington). The relative protein concentration was then calculated as the ratio of the sum of the areas of the transitions of the endogenous (light) peptide divided by the sum of the areas of the transitions of the corresponding heavy peptide. This ratio was then log-transformed. Noisy transitions (signal-to-noise <3) were not included in the quantification.

To ensure the best possible instrument performance, the reproducibility of the analysis platform (HPLC, source, MS) was checked daily using a reference protein mixture consisting of six *Bos taurus* proteins (MICHROM, USA), and cleaning and calibration of the instrument was performed as needed. Coefficients of variation for peak areas and retention times for technical replicates were less than 10% for the same samples run on different days and less than 5% for the same samples run on the same day. No instrumental drift was observed during sample analysis.

Except for RUNX2, which was only measured in the primary adipocyte samples, the peptides and proteins presented in this paper were detected and measured in all samples and in all replicates. All data, even outliers, were included in the analysis. Normalization was not carried out across samples. Instead, each sample was normalized to a control sample prepared in parallel on the same day under exactly the same conditions. Each sample and the corresponding control sample were then analyzed on the mass spectrometer back-to-back on the same day.

### Immunocytochemistry

Following procedures described in (9), preadipocyte cells were plated at 15,000 cells/well and treated as indicated in 96-well plates (Costar). Cells were fixed with 3% paraformaldehyde in PBS for 30 min. Then the cells were gently washed three times with PBS and permeabilized with 0.05% saponin (Sigma #47036), blocked with 3% BSA (Sigma #7906), and stained with DAPI (1:10,000), anti-PPARG (1:500, Santa Cruz Biotech #sc-7273), anti-CEBPA (1:500, Santa Cruz Biotech #sc-61 or #sc-7962), anti-CEBPB (1:500, Santa Cruz Biotech #sc-150), anti-pAKT (Ser473, Cell Signaling #D9E), or BODIPY 493/503, (1 μg/ml, Molecular Probes #D-3922). Alexa Fluor-514 (#A31558), -555 (#A21429), -594 (#A11032), and -647 (#A31571) (1:1,000, Invitrogen) were used as secondary antibodies. Images were taken using an ImageXpress Micro XL system (Molecular Devices) and analyzed using Cell Profiler software.

### siRNA synthesis and transfection

siRNA for each target protein was made using Giardia dicer as described previously (9). Regions (∼500–600 bp) from each gene were PCR amplified from a mouse cDNA library, and DNA was reverse transcribed using T7 RNA polymerase. dsRNA molecules were then diced with Giardia dicer and purified on glass fiber columns using RNA PureLink RNA lysis buffer and wash buffer II (Life Technologies). siRNA concentration was determined using a NanoDrop spectrophotometer (Thermo Scientific), and OP9 cells were transfected in 96-well plates with 20 nM siRNA using Lipofectamine RNAiMAX (Life Technologies) in MEMα with 20% FBS and 100 U/ml penicillin/streptomycin following the company’s protocol. Primer sequences used to make siRNA are listed in supplementary Table 2.

## RESULTS

### Development of a targeted proteomics approach to obtain quantitative nuclear protein profiles from primary mouse adipocytes and SVCs

**Fig. 1.**
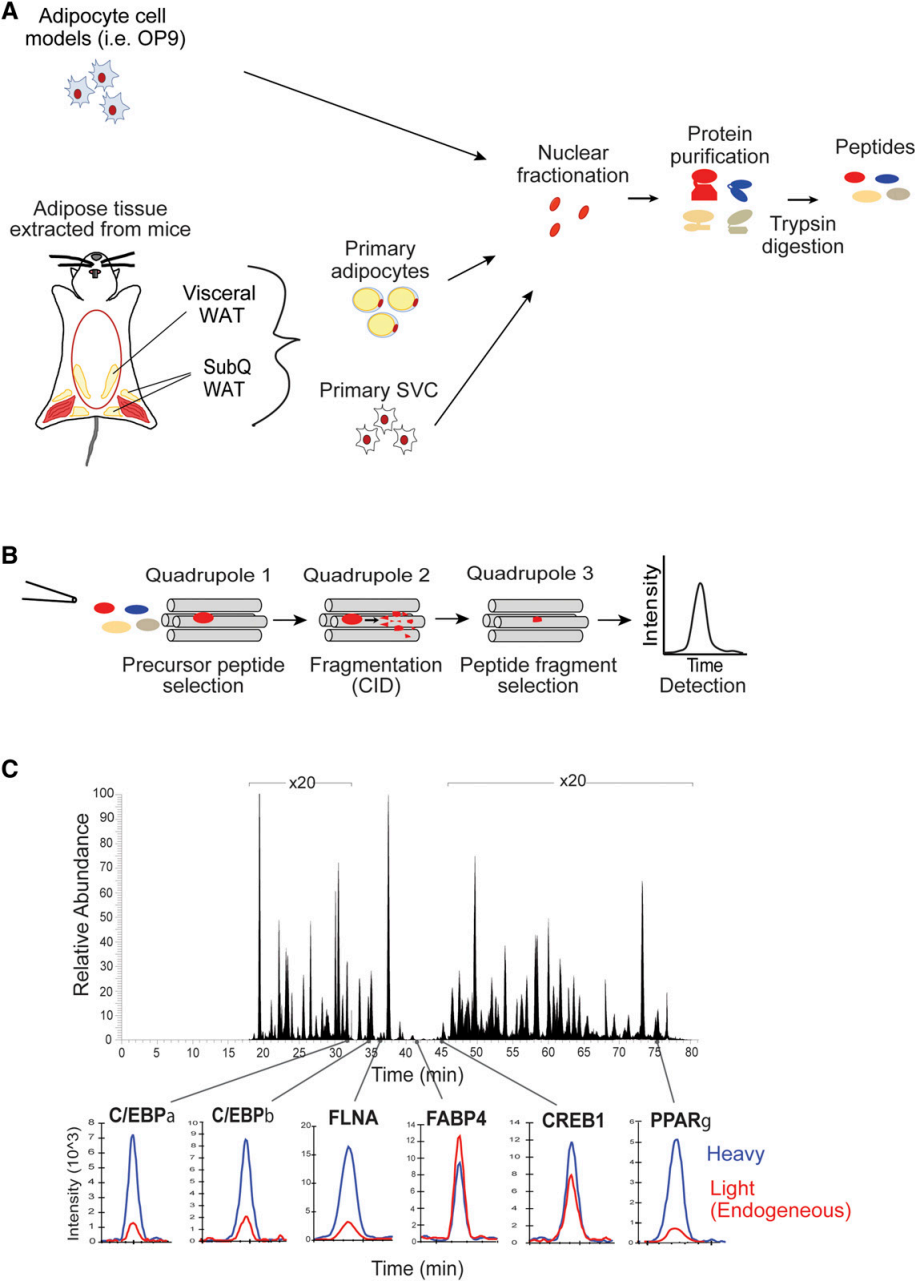
Development of a method to quantitatively profile nuclear protein concentrations in different adipose depots in insulin-resistant versus control mice. A: Adipose tissues were digested with collagenase and separated into mature adipocytes and SVCs. Nuclear proteins were isolated from cells and digested using trypsin. B: To carry out SRM-MS analysis, the samples were injected into a triple-quadrupole mass spectrometer, and the intensity of specifically targeted peptides was measured over time. C: Total ion chromatogram of adipocytes isolated from C57BL/6J mice. Heavy peptides (internal standards) were added to each sample at a known concentration. The heavy peptides (blue) and light endogenous peptides (red) eluted at the same time. The ratio of the light to heavy peptides was calculated to determine relative abundance of each peptide.

Building on a proteomics approach we developed to quantitatively measure the expression of nuclear proteins in adipocyte cell culture models, we now adapted it for use in adipocytes and SVCs from different WAT depots in mice (**Fig. 1A**) (10). WAT was excised from visceral (epidydimal) and subcutaneous fat depots in mice and digested with collagenase to separate out adipocytes and SVCs. Cells were broken using mechanical douncing, and nuclei were isolated from the resulting lysate. To isolate nuclei from the primary adipocytes, it was essential to spin the lysate through a sucrose gradient in order to remove the copious fat in the cells. The nuclei were broken using a high salt solution, and proteins were extracted, digested into peptides, and prepared for MS analysis.

Our approach is based on SRM-MS, which uses a triple quadrupole mass spectrometer to target and detect the same set of peptides repeatedly in different samples (Fig. 1B). SRM-MS has been shown to be an effective approach to quantitate targeted proteins in differentially perturbed states (10–14). In this study, we selected the proteins to be measured based on siRNA screening results and published literature that supported a role for each protein in adipocyte function (15, 16). To quantitatively measure the abundance of each peptide, heavy peptides corresponding to target peptides were spiked into each sample at a known concentration. By calculating the ratio of light endogenous peptide to the heavy peptide, the relative abundance of each protein was quantified. As shown by the example chromatogram of adipocytes isolated from visceral fat from a control C57BL/6J mouse (Fig. 1C), our method has the sensitivity to quantify low abundant proteins, including transcription factors such as PPARG, CEBPB, and CREB1. Finally, unlike RNA microarrays, SRM-MS quantifies protein abundance, thereby overcoming discrepancies between RNA and protein levels (17). The proteins and peptides measured in this study are listed in supplementary Table 1.

**Fig. 2.**
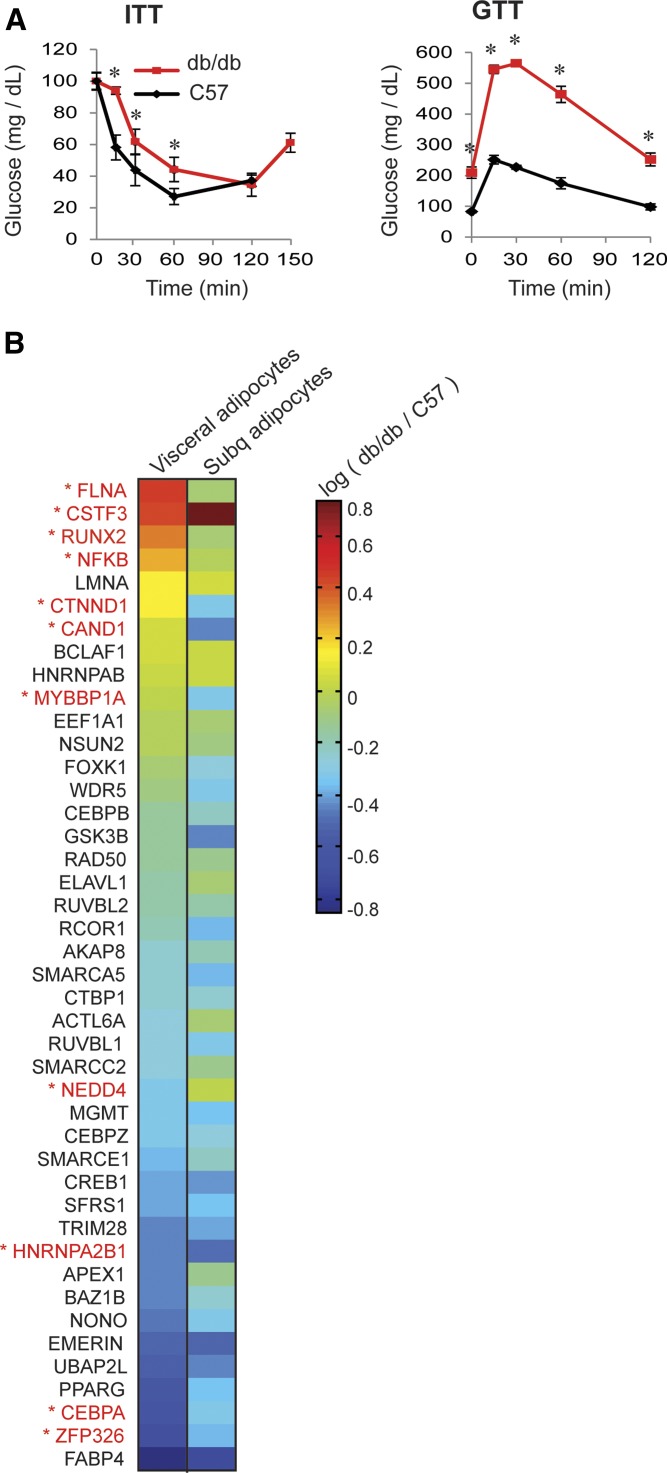
Nuclear protein expression levels are changed differently in visceral and subcutaneous adipocytes in insulin-resistant mice. A: The insulin sensitivities of 10-week-old db/db and C57BL/6J mice were determined by ITT and GTT. Blood glucose levels subsequent to intraperitoneal injection of insulin or glucose were monitored for the indicated times. Error bars show SEM. The ITT was carried out on nine db/db and eight C57 mice. The GTT was carried out on six db/db and four C57 mice. *P < 0.05 determined by Student’s *t*-test. B: Heat map showing changes in protein expression in visceral and subcutaneous fat in insulin-resistant mice plotted on a log-scale. Each value represents the average of three experiments (biological replicates). Each experiment used adipose tissue from either six C57BL/6J or two db/db mice. See supplementary Fig. 2 for bar plots with error bars. The proteins that are most differentially expressed between visceral and subcutaneous insulin-resistant adipocytes are marked in red.

We used our approach to profile nuclear proteins from insulin-resistant adipocytes excised from epididymal visceral WAT and inguinal subcutaneous WAT from 10-week-old db/db mice, a commonly used insulin-resistant mouse model, and from C57BL/6J (control) male mice. To verify that the db/db mice were insulin resistant, we assessed systemic insulin sensitivity with GTTs and ITTs (**Fig. 2A**). Our results showed that the db/db mice were hyperglycemic and showed delayed and reduced glucose clearance, indicating these mice were insulin resistant and glucose intolerant.

The heat map in Fig. 2B shows how protein expression levels were altered under the insulin-resistant state in visceral versus subcutaneous adipocytes in an average of three biological replicate experiments. For each experiment, adipocytes were isolated from visceral and subcutaneous fat excised from either six C57BL/6J or two db/db mice, and thus a total of 18 control animals and 6 db/db mice were used to generate the three biological replicates. Values above 0 indicate upregulation, while values below 0 indicate downregulation of protein abundances in db/db versus C57BL/6J mice. Fold changes with error bars are plotted in supplementary Fig. 2. From this data, we identified proteins whose abundance was significantly altered by insulin resistance in visceral versus subcutaneous adipocytes.

### Almost all of the most differentially abundant proteins in visceral versus subcutaneous insulin-resistant adipocytes regulate adipogenesis

**Fig. 3.**
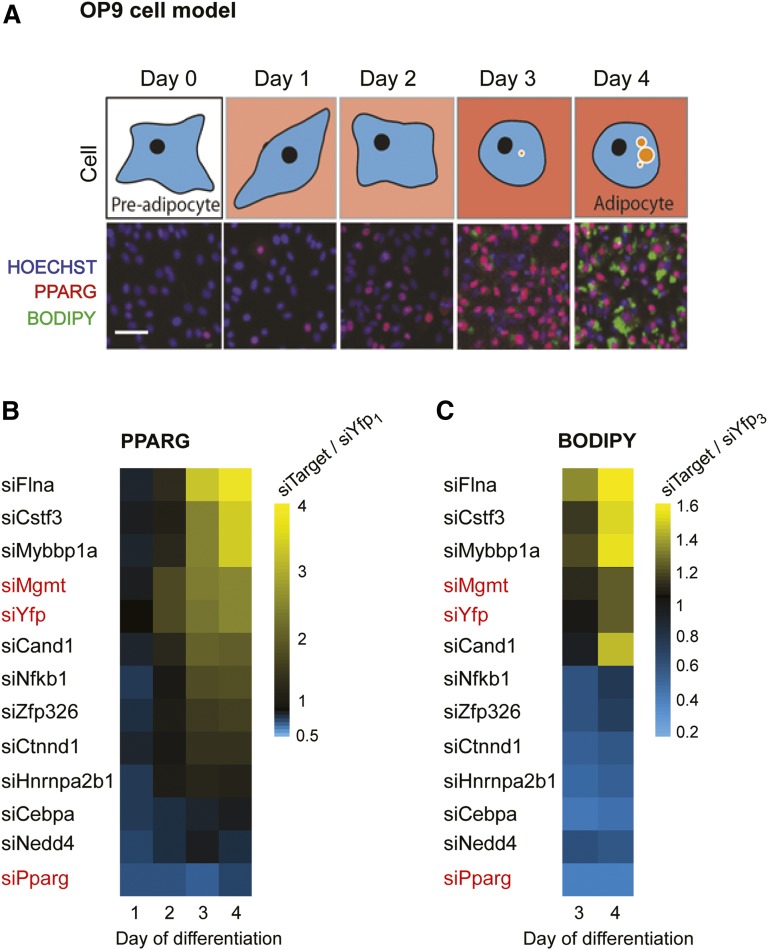
The proteins that are most differentially expressed between insulin-resistant visceral and subcutaneous fat affect adipogenesis. A: OP9 cells were differentiated over 4 days in culture following a standard protocol. Images for BODIPY- and PPARG-stained cells are shown at different time points during differentiation. Scale bar represents 40 μm. B, C: OP9 cells were transfected on day 0 with the indicated siRNA and differentiated in culture. siRNA targeting Yfp was used as a negative control, and siRNA targeting Pparg was used as a positive control. B: Cells were fixed at days 1–4 during differentiation and stained for PPARG intensity. Each datapoint (square) on the heatmap represents the average PPARG intensity in ∼15,000 cells transfected with the indicated target siRNA (siTarget) divided by the average PPARG intensity in ∼15,000 cells transfected with control siRNA targeting YFP and fixed at day 1 or 24 h after transfection (siYFP_1_). C: Cells were fixed at days 3 and 4, and stained for BODIPY to assess lipid content. Each datapoint on the heatmap represents the average BODIPY intensity in ∼15,000 cells transfected with the indicated target siRNA (siTarget) divided by the average BODIPY intensity in ∼15,000 cells transfected with control siRNA targeting YFP and fixed at day 3 or 72 h after transfection (siYFP_3_).

Dysfunctional adipocytes could originate from defects in the differentiation process resulting in immature adipocytes that lack proper adipocyte functions. To understand whether the proteins whose differential abundance distinguished insulin-resistant visceral from subcutaneous WAT might be causing defects in differentiation, we carried out in vitro differentiation experiments using siRNA knockdowns of the proteins whose expression levels were the most changed in visceral compared with subcutaneous insulin-resistant adipocytes (indicated in red). OP9 cells, a mouse cell line that shows many of the same adipogenic attributes as preadipocytes in vivo (8), were transfected with siRNA targeting each protein, and differentiated in cell culture. Cells were stained for PPARG levels and lipid content (using BODIPY) at different time points during the 4 days required for OP9 cell differentiation (**Fig. 3A**). Figure 3B, C shows heat maps of fold-changes in PPARG and BODIPY intensities for OP9 cells treated with the different siRNA. siRNA targeting YFP and PPARG (marked in red) were used as controls. Because PPARG and lipid content increase over the 4 day time course of differentiation (9), both PPARG and BODIPY intensities can be seen to increase for the siYFP control (Fig. 3B, C).

As expected, cells treated with siRNA targeting PPARG, the master regulator of adipogenesis, were unable to differentiate and failed to accumulate lipid, as shown by a lack of BODIPY staining (Fig. 3C). The 11 most differentially abundant proteins in visceral versus subcutaneous insulin-resistant adipocytes are marked in red in Fig. 2B. Figure 3B and C show that siRNA-mediated depletion of 9 out of 10 of these proteins had significant effects on adipogenesis. In addition, a previous study showed, by siRNA-mediated depletion, that Runx2 is a negative regulator of adipogenesis (10). In the experiments shown in Fig. 3, depletion of Nfκb, Zpf326, Ctnnd1, Hnrnpa2b1, Cebpa, and Nedd4 decreased both BODIPY and PPARG compared with the Yfp control, suggesting that these proteins are positive regulators of adipogenesis. On the other hand, depletion of Flna, Cstf3, and Mybbp1a increased PPARG expression levels and lipid accumulation, suggesting that these proteins are negative regulators of adipogenesis. As a control, siRNA-mediated depletion of Mgmt, a protein whose expression was not significantly altered by IR in visceral versus subcutaneous adipocytes, did not affect adipogenesis.

**Fig. 4.**
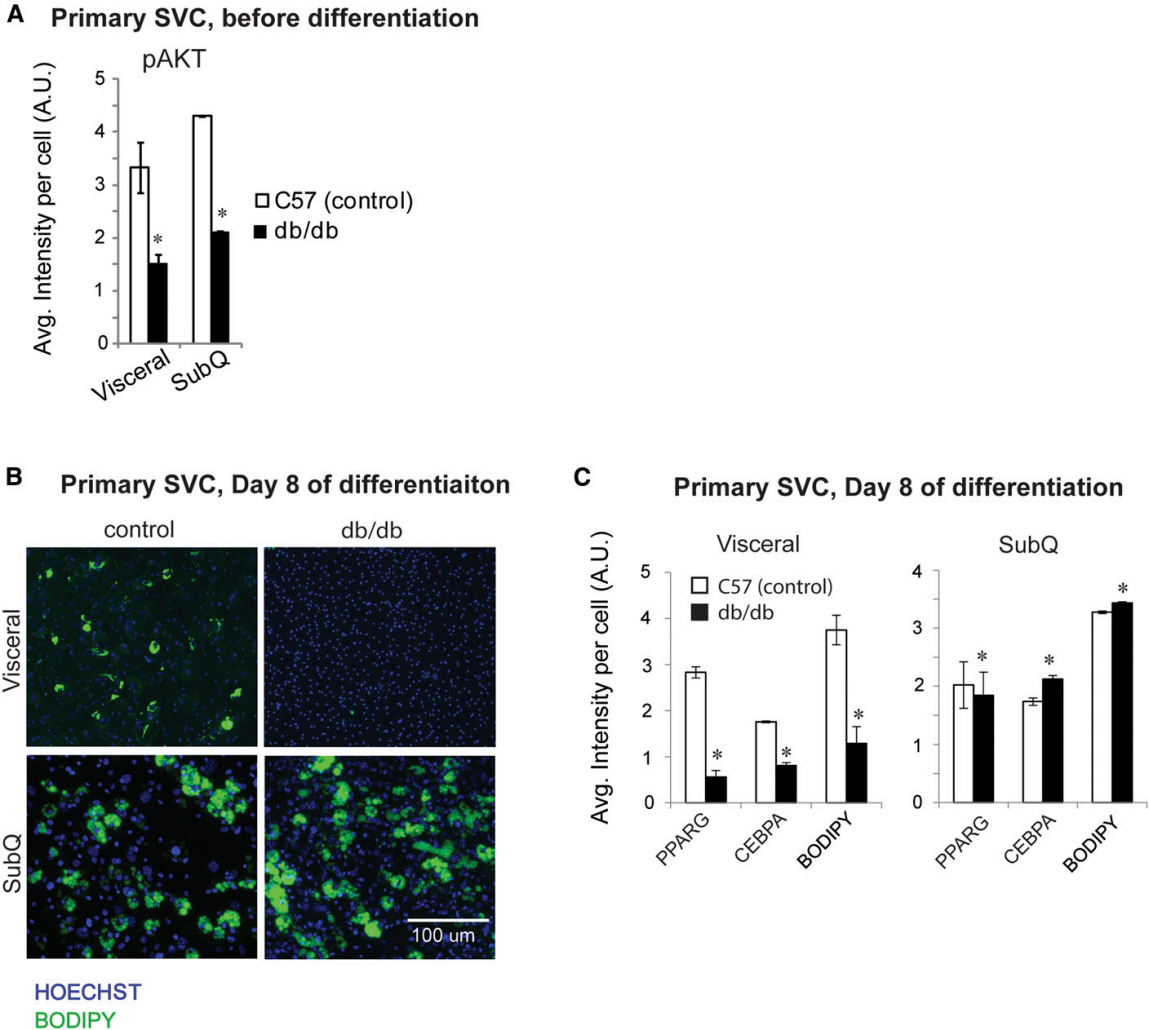
Immunocytochemistry analysis indicates that visceral, but not subcutaneous, SVCs are defective in adipogenesis. A: SVCs isolated from visceral and subcutaneous adipose tissues of db/db and C57BL/6J mice were cultured and analyzed by immunocytochemistry using anti-pAKT. Intensity levels in response to insulin were quantified and plotted in a bar graph. For each biological replicate, ∼5,000 cells were analyzed. B: SVCs from visceral and subcutaneous adipose tissues were differentiated over 8 days in culture. Cells were stained with BODIPY and Hoescht. C: On day 8, cells were also stained for PPARG and CEBPA, and intensities were quantified. For each biological replicate, ∼8,000 cells were analyzed. Error bars indicate SEM (n = 3 biological replicates). *P < 0.05 determined by the Student’s *t*-test.

To determine whether the differentiation capacity of SVCs is affected in insulin-resistant visceral and subcutaneous tissues, SVCs from the visceral and subcutaneous tissue depots of db/db and C57BL/6J mice were differentiated in culture. Although both depots demonstrated impaired insulin sensitivity at the cellular level, as determined by levels of pAKT (**Fig. 4A**), subcutaneous SVCs from db/db mice showed no impairment in differentiation compared with control mice, whereas visceral db/db SVCs failed to differentiate (Fig. 4B, C). This lack of differentiation could be partially due to a depletion of the preadipocyte pool, because obesity has been shown to reduce the number of preadipocytes in adipose tissue, for example, by approximately 50% in obese versus lean women (18). However, a preadipocyte pool still exists, even in severely obese individuals (19), and the total lack of differentiation of the visceral db/db SVCs suggested that the preadipocyte pool was defective in differentiation capacity.

### A combination of inflammatory and high-fat stimuli best mimics the protein abundance profile in db/db visceral fat

**Fig. 5.**
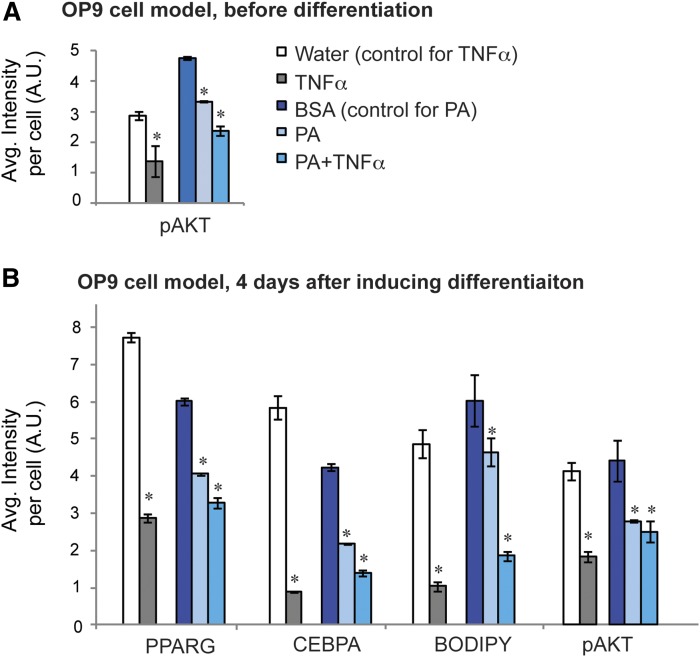
TNFα and PA induce IR and defective differentiation in cell culture. A: OP9 preadipocytes were treated with TNFα, PA, or both TNFα and PA for 24 h. Water was used as a control for TNFα, and BSA-ethanol (BSA) was used as a control for PA and PA+TNFα treatments. Insulin sensitivity was determined by staining cells for pAKT by immunocytochemistry. Intensities were quantified and plotted on a bar graph. B: To determine the effect of different treatments on differentiation, OP9 cells were differentiated in the presence of TNFα, PA, or both TNFα and PA. Cells were stained for PPARG, CEBPΑ, BODIPY, and pAKT on day 4, and intensities were quantified. Error bars indicate SEM (n = 3 biological replicates). For each biological replicate, ∼15,000 cells were analyzed. *P < 0.05 determined by the Student’s *t*-test.

To explore what might be causing the defective differentiation in db/db visceral SVCs, we compared nuclear protein profiles to protein profiles obtained by apply­ing different IR-inducing stimuli to OP9 cells. We first checked that the different IR-inducing stimuli resulted in preadipocytes that were both insulin resistant and defective in adipogenesis, similar to the characteristics of the db/db visceral preadipocytes. OP9 preadipocytes treated for 24 h with TNFα, free fatty acids in the form of PA, or a combination of TNFα and PA were all insulin resistant, as shown by the decreased levels of pAKT (**Fig. 5A**). All of these models also showed decreased levels of PPARG and CEBPA (Fig. 5A), which is consistent with other studies of insulin-resistant cells in vitro (20). To determine whether TNFα and PA impede adipogenesis, OP9 cells were differentiated in the presence of TNFα, PA, both PA and TNFα, or ethanol-BSA complex (control) and assessed for adipocyte markers at day 4 (Fig. 5B). The results show TNFα- and PA-treated cells both inhibited adipogenesis, indicated by reduced PPARG expression levels. The higher level of BODIPY staining in PA-treated cells compared with TNFα is most likely due to the uptake of PA into the cells.

**Fig. 6.**
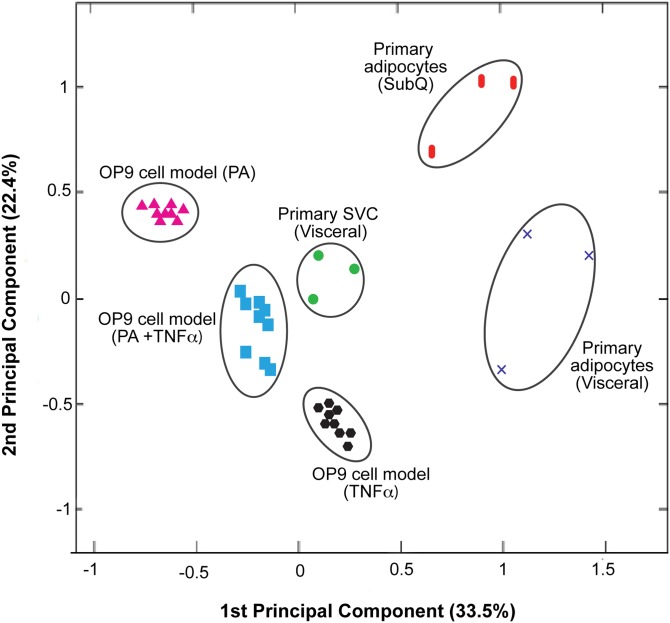
Principal component analysis shows that the protein profiles of insulin-resistant primary adipocytes, primary SVCs, and OP9 cell culture models are distinct based on changes in protein abundances. The proximity of the clusters indicates how similar the samples are, and interestingly, the protein profile of visceral SVCs most closely matches that of OP9 preadipocytes treated with both PA and TNFα. Supplementary Fig. 6 shows how the different proteins contribute to the first two principal components.

To compare nuclear protein abundance across these varied models of IR, we purified nuclear proteins from the insulin-resistant cell culture models and the db/db visceral SVCs, prepared samples, and analyzed them using SRM-MS. **Figure 6** shows a principal component analysis of the nuclear protein profiles from the different primary cell and cell model experiments. The distinct clustering shows that these cell types are all discrete from each other. Interestingly, the db/db visceral SVC population correlates most closely with the OP9 cells treated with PA and TNFα. The fold changes for the measured nuclear proteins in cells treated with PA and TNFα compared with control and db/db visceral SVCs are shown in supplementary Figs. 3–5. Examining how each protein contributes to the first two principal components (plotted in supplementary Fig. 6) shows the two proteins that contributed most to the first principal component were CSTF3 and FLNA, both strong negative regulators of adipogenesis, as shown in the siRNA results in Fig. 3B, C.

## DISCUSSION

Visceral, but not subcutaneous, adipose tissue mass is highly correlated with IR and metabolic diseases (5, 6). Previous studies have identified intrinsic differences among preadipocyte populations isolated from different fat depots using several different measurement techniques (21–23). Here we developed a targeted proteomics method using SRM-MS to quantitatively measure nuclear protein abundances in mature adipose tissue excised from mice, and have applied this method to identify differentially expressed proteins between visceral and subcutaneous adipocytes from db/db insulin-resistant mice and C57BL/6J control mice. We showed that nuclear protein abundance levels are not uniformly altered in subcutaneous and visceral adipocytes in insulin-resistant db/db mice, and that many of the most differentially expressed proteins affected adipogenesis. This observation is in line with previous studies in which gene expression levels of adipogenic genes such as Pparg and Cebpa were decreased in IR in both humans and mice (24, 25).

Here we observed that proteins we found to be negative regulators of adipogenesis from our siRNA experiments (Fig. 3), such as FLNA, CSTF3, and MYBBP1A, were consistently increased in visceral versus subcutaneous db/db adipocytes. On the other hand, the proteins that we found to be the most decreased in visceral versus subcutaneous db/db adipocytes, such as HNRNPA2B1, NEDD4, CEBPA, and PPARG, were positive regulators of adipogenesis. These results suggest that a key difference between visceral and subcutaneous insulin-resistant (db/db) adipocytes is defective differentiation.

The likelihood of defective differentiation being a main difference between visceral and subcutaneous insulin-resistant adipose tissue was further supported by a reduced differentiation capacity of visceral SVCs isolated from db/db mice compared with the SVCs isolated from C57BL/6J control mice, while subcutaneous SVCs from db/db mice retained their capacity to differentiate in vitro. Previous clinical studies have shown decreased adipogenesis in subcutaneous adipose tissue of insulin-resistant patients, but this observation was not made with our subcutaneous SVCs isolated from db/db mice (26). In summary, our results suggest that insulin-resistant visceral adipose tissue is defective in differentiation compared with insulin-resistant subcutaneous adipose tissue. This defective differentiation would result in a lack of mature adipocytes that are capable of uptaking glucose in response to insulin, possibly explaining why visceral tissue is more closely associated with systemic IR.

We also observed increased levels of pro-inflammatory proteins such as NFκB1, as well as CEBPB, in visceral db/db adipocytes. The increased levels of both inflammation and CEBPB have been observed in many other studies in both cell culture and in animals that show TNFα-induced inflammation and IR (20, 27, 28). To start to understand what might be causing the defective adipogenesis observed in the db/db visceral adipose tissue, we used our SRM-MS method to compare the abundance of a group of nuclear proteins in different adipocytes from db/db mice to the nuclear protein profiles observed in different cell culture models of IR. We showed that the nuclear protein profile of visceral SVCs correlates more with the nuclear protein profile of an in vitro model of insulin-resistant cells treated with both TNFα and PA, suggesting that the defective adipogenesis in db/db visceral tissue could be due to a combination of inputs from inflammation and fatty acids.

This study focused on quantifying transcription factors and chromatin remodeling proteins due to their importance during differentiation. However, because insulin signaling pathways involve many membrane proteins and cytosolic proteins, it will be of interest to study changes in proteins in other cellular compartments in the future. Several studies have profiled primary adipocytes from different fat depots using microarrays, but the SRM-MS-based method we developed has the advantage of profiling at the protein level, which is the most direct approach to assess the state of the cell because the expression level and molecular form of proteins is an integration of genomic and environmental factors, and developmental, metabolic, and homeostatic processes. Because proteins mediate cell activities downstream of mRNA, it is essential that protein levels are quantified accurately to characterize molecular differences among different cell populations. Also, many studies have now shown that mRNA expression matches very poorly with protein expression because of variations in protein half-lives, differences in the degree of posttranslational modifications, and other factors (17, 29, 30).

The SRM-MS method we developed can be applied to profile adipose tissues from other animal models and can be used to identify important biomarkers that are commonly associated with impaired differentiation or IR. For instance, IR can be observed in high-fat diet-fed mice, aged mice, or different genetic mouse models. It is likely that insulin-resistant adipocytes from different animal models have different molecular features. Using SRM-MS, it will be possible to determine how these cells differ, or to study the effects of drugs and small molecules on the expression levels of several tens to hundreds of proteins from a single sample. These future studies will advance our understanding of molecular mechanisms underlying the development of IR, and will aid in the development of therapeutics to help prevent more severe diseases. Finally, our protocols and approach can easily be extended to humans and other organisms, and to other tissues.

Here we have introduced a method to quantitatively measure nuclear protein abundances from different adipose depots in different mouse models and have applied it to control C57BL/6J and insulin-resistant db/db mice. Whereas db/db mice are commonly used as a model for IR, it is plausible that leptin deficiency might be contributing to the differences we observed. In the future, to distinguish between differences due to IR versus leptin deficiency, it would be interesting to apply our method to other mouse models of IR, such as diet-induced obesity.

In summary, this study describes a novel protein profiling method that we developed to quantify large numbers of key, but low-abundant, nuclear proteins in primary fat cells which regulate whole body metabolism and play a central role in metabolic disease. We applied our method to quantitate and compare for the first time the endogenous abundance of more than 40 transcription factors, chromatin-remodeling, and other key nuclear proteins in different adipose depots in insulin-resistant mice without needing antibodies and requiring only 2 μg of protein to analyze each sample, approximately 20 times less than is required for a Western blot lane. This method allowed us to uncover new candidate proteins involved in IR that could be potential drug targets, as well as to understand fundamental differences between insulin-resistant and insulin-sensitive adipocytes in vivo at the protein level. This method will likely have broad utility in understanding the origin of adipocyte dysfunction in other mouse models of IR, as well as in humans.

## Supplementary Material

Supplemental Data
