# Supplementary material for: Using SRM-MS to quantify nuclear protein abundance differences between adipose tissue depots of insulin-resistant mice
Source: J Lipid Res. 2015 May;56(5):1068–78. doi: 10.1194/jlr.D056317 (PMC4409283; doi:10.1194/jlr.D056317)
Supplement: Supplemental Data [file supp_56_5_1068__index.html]

Using SRM mass spectrometry to quantify nuclear protein abundance differences between adipose tissue depots of insulin-resistant mice — Using SRM-MS to quantify nuclear protein abundance differences between adipose tissue depots of insulin-resistant mice — Supplemental Data 

# Using SRM-MS to quantify nuclear protein abundance differences between adipose tissue depots of insulin-resistant mice

## Supplemental Data

**Files in this Data Supplement:**

- Supplemental Figure S1 - Verifying the specificity of the subcellular fractionation approach
- Supplemental Figure S2 - Changes in nuclear protein levels measured using SRM-MS in primary adipocytes isolated from db/db (insulin-resistant) and C57Bl6 (control, insulin-sensitive) mice.
- Supplemental Figure S3 - Changes in nuclear protein levels measured using SRM-MS in OP9 cell model preadipocytes made insulin-resistant with TNF&#x26;#945; and/or palmitate.
- Supplemental Figure S4 - Changes in nuclear protein levels measured using SRM-MS in primary visceral SVC isolated from db/db (insulin-resistant) and C57Bl6 (control, insulin-sensitive) mice.
- Supplemental Figure S5 - The proteins that are common to visceral SVC and to PA+TNF&#x26;#945; treated OP9 cells could be key proteins that are changed in insulin-resistant SVC in vivo. This heat map is a side-by-side comparison of the same data as is plotted in Supp. Figs. S3-S4. Protein abundances were quantified for visceral SVC from db/db versus C57Bl6 (control) mice, as well as from OP9 cells treated with TNF&#x26;#945;, PA or both PA and TNF&#x26;#945; for 24 hours versus untreated (control) OP9 cells. Changes in protein abundances (log-scale) resulting from each insulin-resistant condition ratioed over the control measurements are shown in a heat map. Proteins highlighted in red indicate proteins that are the most similarly changed in db/db visceral SVC compared with OP9 cells treated with both PA and TNF&#x26;#945;. Each datapoint (square) on the heat map represents the average of 3 biological replicates (for SVC measurements) or 9 biological replicates (for OP9 cell measurements).
- Supplemental Table S1 - List of measured peptides and peptide fragments (transitions)
- Supplemental Table S2 - List of primers used to make siRNA
- Supplemental Figure 6 - Relative contributions of each protein to Principal Components #1 and #2.
- Supplemental Table S3 - Relative protein abundance values for the 42 monitored proteins measured for 6 types of experiments: 1) 3 replicates of db\_visceral: visceral adipocytes from db/db versus C57Bl6 (control) mice, 2) 3 replicates of db\_subQ: subcutaneous adipocytes from db/db versus C57Bl6 (control) mice, 3) 3 replicates of db\_SVC: stromal vascular cells (SVC) from db/db versus C57Bl6 (control) mice, 4) 9 replicates of TNF: OP9 cells treated with TNFa for 24 hours ratioed over untreated OP9 cells, 5) 9 replicates of PA\_TNF: OP9 cells treated with TNFa and palmitate (PA) for 24 hours ratioed over untreated OP9 cells, and 6) 9 replicates of PA: OP9 cells treated with palmitate (PA) for 24 hours ratioed over untreated OP9 cells. The values listed are the log of the ratio (insulin-resistant or treated / control).
