# Supplementary material for: Using SRM-MS to quantify nuclear protein abundance differences between adipose tissue depots of insulin-resistant mice
Source: J Lipid Res. 2015 May;56(5):1068–78. doi: 10.1194/jlr.D056317 (PMC4409283; doi:10.1194/jlr.D056317)

Supplementary Figure S1. Verifying the specificity of the subcellular fractionation approach. Two nuclear preparations were used: 1) a OP9 cell nuclear prep and 2) a primary adipocyte nuclear prep. To isolate nuclei from OP9 cells, OP9 cells were dounced through 30G needles to break open the cells and then the lysate was spun down to pellet down the nuclei. Primary adipocytes contained so much fat that the sucrose gradient was needed to remove the excess fat from the nuclei. Thus, to isolate nuclei from primary adipocytes, cells were dounced in a glass-glass dounce homogenizer to break open the cells and then the lysate was spun through a sucrose gradient (0.5 to 1.25 M) to isolate nuclei.

To verify the specificity of the subcellular fractionation approach, 3 μg of each fraction was analyzed using SRM-MS while monitoring the following proteins in each sample: Tubulin beta-5 (control cytosolic protein) and HNRNPA2B1 (control nuclear protein). The cytosolic fraction shown is the supernatant obtained when spinning the OP9 cell lysate to pellet out the nuclei. As shown below, both the OP9 cell nuclear prep and the primary adipocyte nuclear prep yielded fractions that were highly specific for nuclear proteins as shown by the fact that control nuclear proteins such as Hnrnpa2b1 were more than 25-fold enriched versus the cytosolic fraction whereas cytosolic control proteins such as Tubulin beta-5 were more than 25-fold decreased. Error bar indicates SEM (n=3 biological replicates)

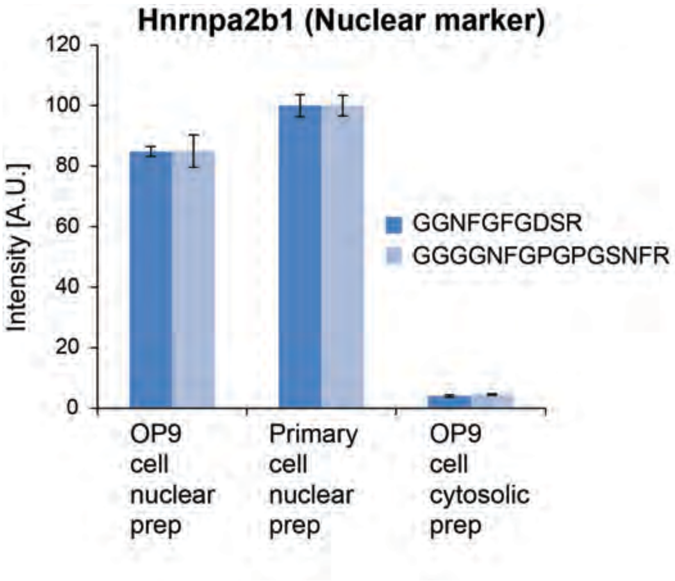

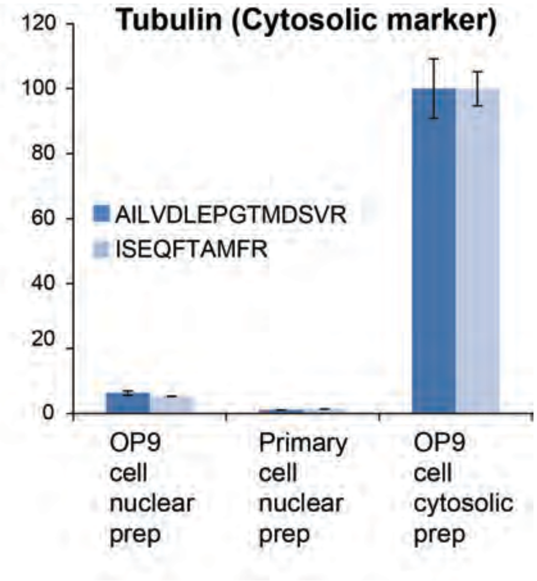

Supplement: Supplemental Data [file supp_D056317_jlr.D056317-1.pdf]
