# Supplementary material for: Using SRM-MS to quantify nuclear protein abundance differences between adipose tissue depots of insulin-resistant mice
Source: J Lipid Res. 2015 May;56(5):1068–78. doi: 10.1194/jlr.D056317 (PMC4409283; doi:10.1194/jlr.D056317)

<u>Supplementary Figure S2:</u> Changes in nuclear protein levels measured using SRM-MS in primary adipocytes isolated from db/db (insulin-resistant) and C57Bl6 (control, insulin-sensitive) mice. Changes in **(A)** visceral adipocytes and **(B)** subcutaneous adipocytes are plotted as the log of (db/db /control). Error bar indicates SEM (n=3 biological replicates). **(C)** Plot of difference between visceral and subcutaneous values.

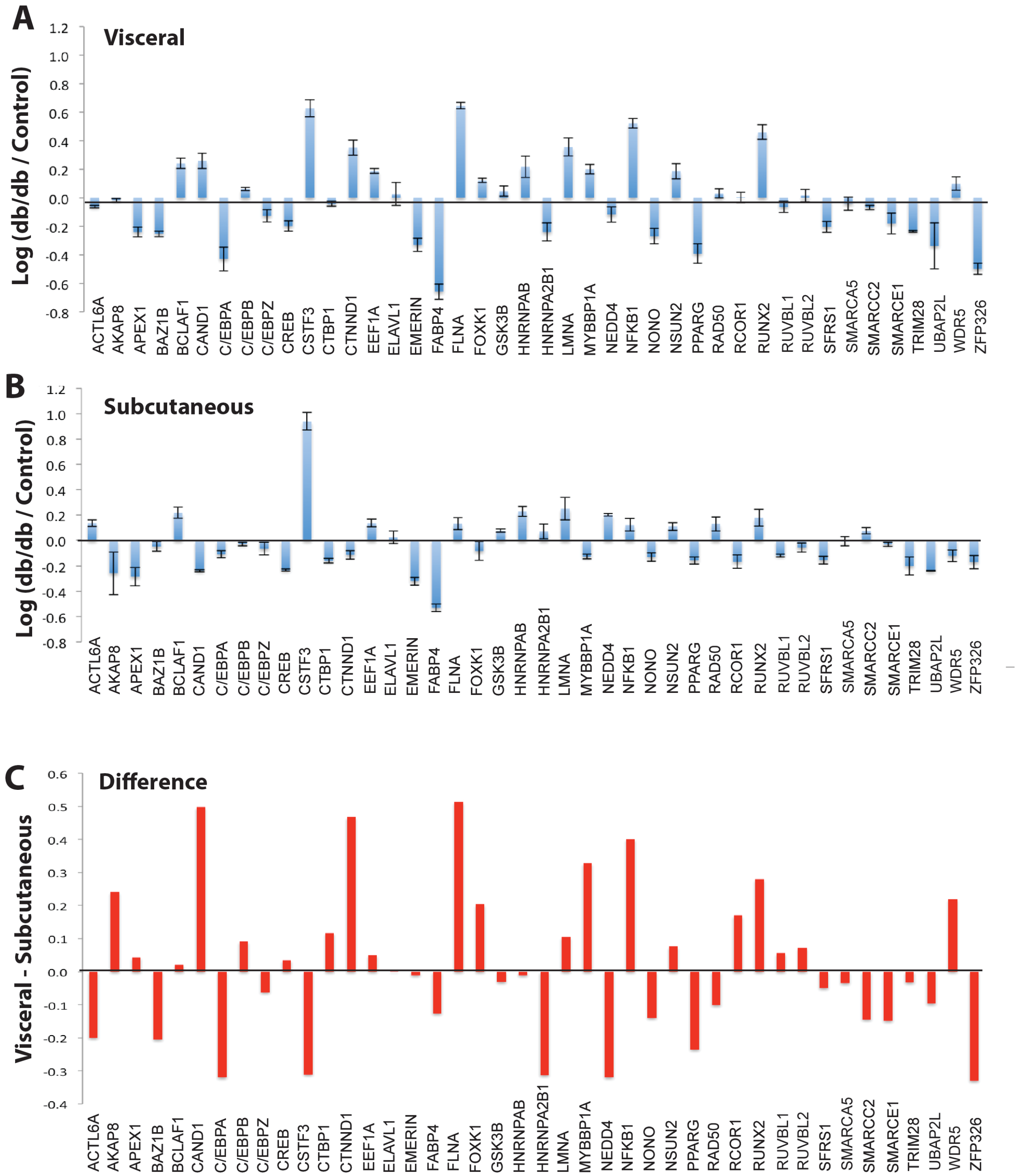

Supplement: Supplemental Data [file supp_D056317_jlr.D056317-2.pdf]
