# Supplementary material for: Using SRM-MS to quantify nuclear protein abundance differences between adipose tissue depots of insulin-resistant mice
Source: J Lipid Res. 2015 May;56(5):1068–78. doi: 10.1194/jlr.D056317 (PMC4409283; doi:10.1194/jlr.D056317)

Supplementary Figure S3: Changes in nuclear protein levels measured using SRM-MS in OP9 cell model preadipocytes made insulin-resistant with TNFα and/or palmitate. Nuclear protein expression levels were measured for OP9 cells treated with **(A)** 5 ng TNFα, **(B)** 0.5 mM palmitate-BSA complex and **(C)** both 0.5 mM palmitate-BSA complex and 5 ng TNFα for 24 hours. Error bar indicates SEM (n=9 biological replicates).

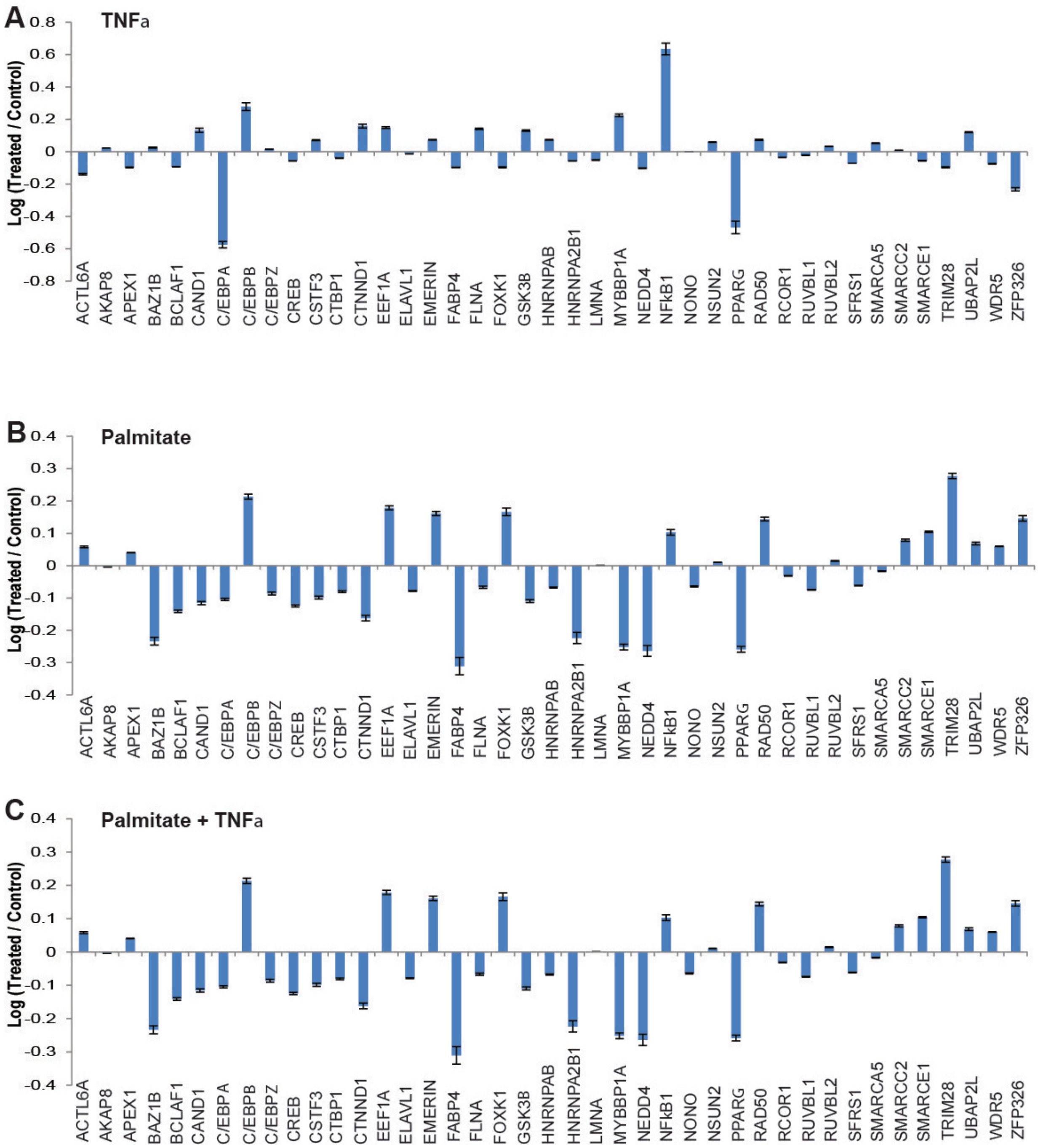

Supplement: Supplemental Data [file supp_D056317_jlr.D056317-3.pdf]
