# Supplementary material for: Using SRM-MS to quantify nuclear protein abundance differences between adipose tissue depots of insulin-resistant mice
Source: J Lipid Res. 2015 May;56(5):1068–78. doi: 10.1194/jlr.D056317 (PMC4409283; doi:10.1194/jlr.D056317)

<u>Supplementary Figure S4:</u> Changes in nuclear protein levels measured using SRM-MS in primary visceral SVC isolated from db/db (insulin-resistant) and C57Bl6 (control, insulin-sensitive) mice. Changes in visceral SVC protein abundances are plotted as the log of (db/db /control). Error bar indicates SEM (n=3).

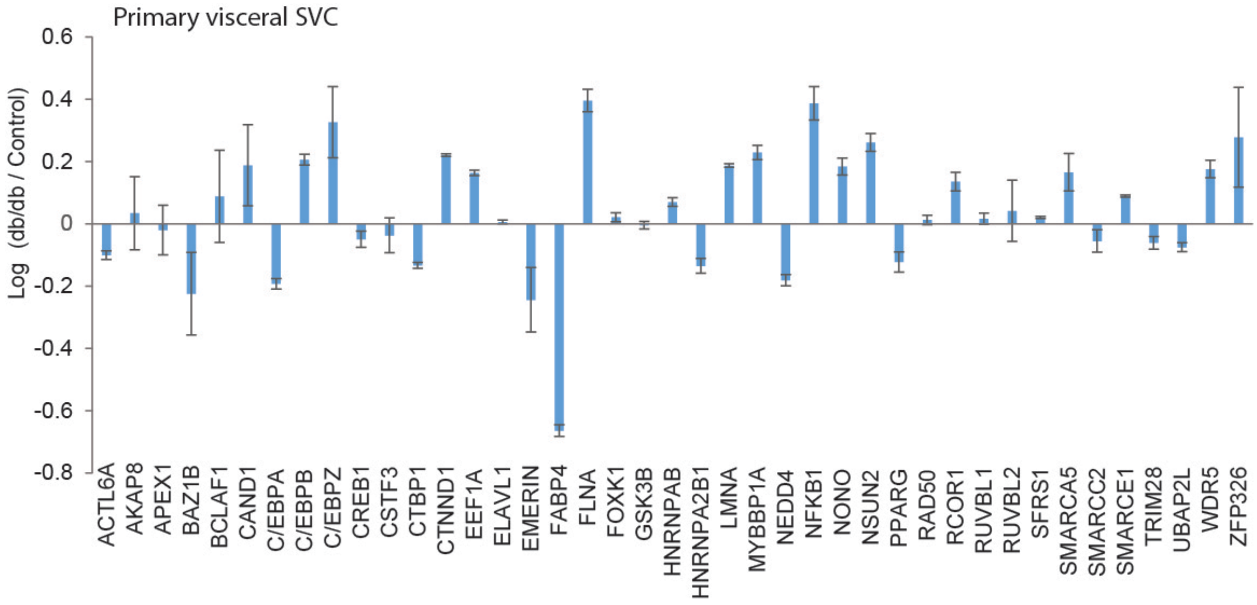

Supplement: Supplemental Data [file supp_D056317_jlr.D056317-4.pdf]
