# Supplementary material for: Using SRM-MS to quantify nuclear protein abundance differences between adipose tissue depots of insulin-resistant mice
Source: J Lipid Res. 2015 May;56(5):1068–78. doi: 10.1194/jlr.D056317 (PMC4409283; doi:10.1194/jlr.D056317)

**<u>Supplementary Figure S5:</u> The proteins that are common to visceral SVC and to PA+TNFα treated OP9 cells could be key proteins that are changed in insulin-resistant SVC in vivo.** This heat map is a side-by-side comparison of the same data as is plotted in Supp. Figs. S3-S4. Protein abundances were quantified for db/db visceral SVC, and OP9 cells treated with TNFα, PA or both PA and TNFα for 24 hours. Changes in protein abundances (log-scale) resulting from each insulin-resistant treatment are shown in a heat map. Proteins highlighted in red indicate proteins that are similarly changed in db/db visceral SVC compared with OP9 cells treated with both PA and TNFα. Each datapoint (square) on the heat map represents the average of 3 biological replicates (for SVC measurements) or 9 biological replicates (for OP9 cell measurements).

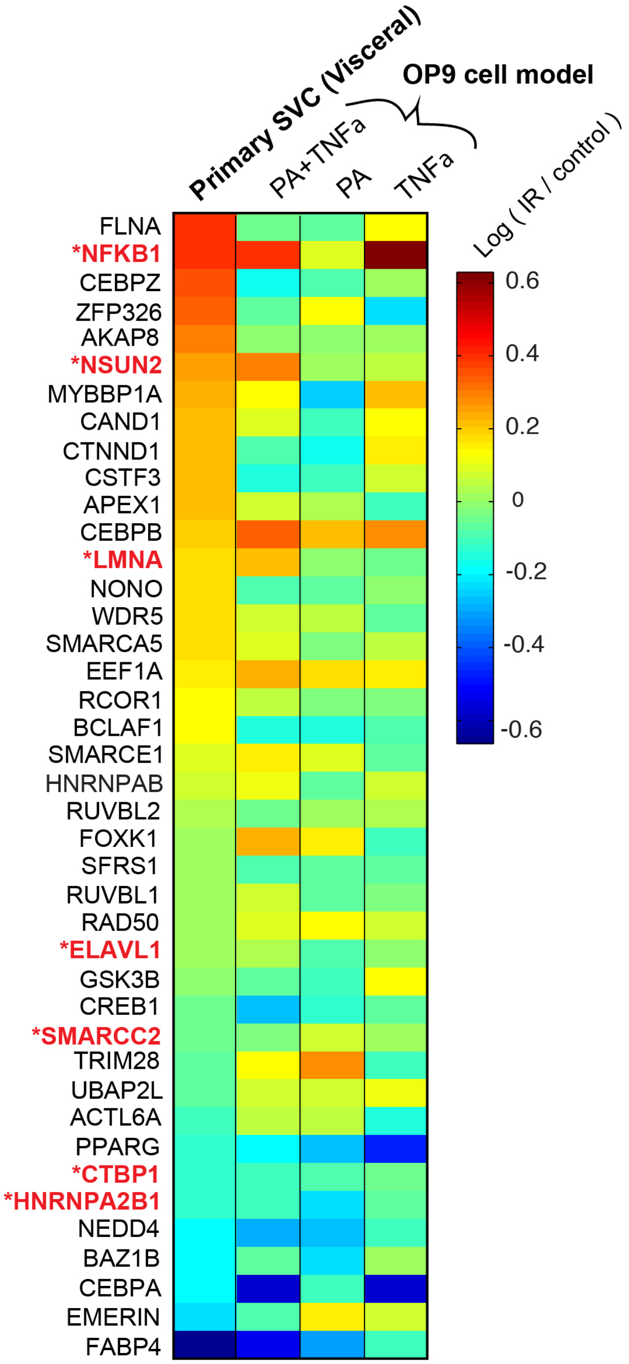

Supplement: Supplemental Data [file supp_D056317_jlr.D056317-5.pdf]
