# Supplementary material for: Using SRM-MS to quantify nuclear protein abundance differences between adipose tissue depots of insulin-resistant mice
Source: J Lipid Res. 2015 May;56(5):1068–78. doi: 10.1194/jlr.D056317 (PMC4409283; doi:10.1194/jlr.D056317)
Supplement: Supplemental Data [file supp_D056317_jlr.D056317-6.pdf]

Protein	UniProt	Peptide	C-Term Isotope	Precursor Mass [m/z]	Precursor Charge	CID [eV]	Fragment Mass [m/z]	Fragment Charge	Fragment Ion
ACTL6A	Q9Z2N8	LIANNTTVER	light	565.811844	2+	19.9	719.368243	1+	y6
							605.325316	1+	y5
							504.277637	1+	y4
			heavy	570.815979	2+	19.9	729.376512	1+	y6
							615.333585	1+	y5
							514.285906	1+	y4
		QGGPTYYIDTNALR	light	784.888812	2+	26.5	965.505071	1+	y8
							802.441743	1+	y7
							689.357679	1+	y6
			heavy	789.892946	2+	26.5	975.51334	1+	y8
							812.450012	1+	y7
							699.365948	1+	y6
		SPLAGDFITMQC[+57.0]R	light	748.355236	2+	25.4	1127.497226	1+	y9
							808.380405	1+	y6
							695.296341	1+	y5
			heavy	753.35937	2+	25.4	1137.505495	1+	y9
							818.388674	1+	y6
							705.30461	1+	y5
AKAP8	Q9DBR0	GGISSGGEGVQDR	light	609.789097	2+	21.2	904.411899	1+	y9
							817.379871	1+	y8
							574.29435	1+	y5
			heavy	614.793232	2+	21.2	914.420168	1+	y9
							827.38814	1+	y8
							584.302619	1+	y5
		LLEEQTC[+57.0]EAASETR	light	818.877784	2+	27.5	1024.4364	1+	y9
							563.278366	1+	y5
							492.241252	1+	y4
			heavy	823.881918	2+	27.5	1034.444669	1+	y9
							573.286635	1+	y5
							502.249521	1+	y4
APEX1	P28352	EEAPDILC[+57.0]LQETK	light	773.376888	2+	26.1	891.460429	1+	y7
							778.376365	1+	y6
							618.345717	1+	y5
			heavy	777.383988	2+	26.1	899.474628	1+	y7
							786.390564	1+	y6
							626.359916	1+	y5
		IC[+57.0]SWNVDGLR	light	610.298047	2+	21.2	946.474105	1+	y8
							859.442077	1+	y7
							673.362764	1+	y6
			heavy	615.302181	2+	21.2	956.482374	1+	y8
							869.450346	1+	y7
							683.371033	1+	y6
		VSYGIGEEEHDQEGR	light	568.920178	3+	23.9	870.370034	1+	y7
							741.327441	1+	y6
							604.268529	1+	y5
			heavy	572.256268	3+	23.9	880.378303	1+	y7
							751.33571	1+	y6
							614.276798	1+	y5
BAZ1B	Q9Z277	YQEITHSIYLAR	light	747.39099	2+	25.3	960.526141	1+	y8
							859.478462	1+	y7
							722.419551	1+	y6
			heavy	752.395125	2+	25.3	970.53441	1+	y8
							869.486731	1+	y7
							732.42782	1+	y6
		FSDFLLDPYK	light	622.813521	2+	21.6	748.423967	1+	y6
							635.339903	1+	y5
							522.255839	1+	y4
			heavy	626.820621	2+	21.6	756.438166	1+	y6
							643.354102	1+	y5
							530.270038	1+	y4

BCLAF1	Q8K019	SPAVTLNER	light	493.766905	2+	17.7	802.441743	1+	y7
							731.404629	1+	y6
							632.336215	1+	y5
			heavy	498.77104	2+	17.7	812.450012	1+	y7
							741.412898	1+	y6
							642.344484	1+	y5
CAND1	Q6ZQ38	AVAALLTIPEAEK	light	663.387385	2+	22.8	900.503674	1+	y8
							787.41961	1+	y7
							573.287868	1+	y5
			heavy	667.394484	2+	22.8	908.517873	1+	y8
							795.433809	1+	y7
							581.302067	1+	y5
		SVILEAFSSPSEEVK	light	811.41961	2+	27.2	862.415253	1+	y8
							775.383225	1+	y7
							688.351196	1+	y6
			heavy	815.42671	2+	27.2	870.429452	1+	y8
							783.397424	1+	y7
							696.365395	1+	y6
CEBPA	P53566	VLELTSDNDR	light	581.290942	2+	20.3	820.379536	1+	y7
							707.295472	1+	y6
							606.247794	1+	y5
			heavy	586.295076	2+	20.3	830.387805	1+	y7
							717.303741	1+	y6
							616.256063	1+	y5
CEBPB	P28033	VLELTAENER	light	587.309134	2+	20.5	961.458515	1+	y8
							832.415922	1+	y7
							719.331858	1+	y6
			heavy	592.313269	2+	20.5	971.466784	1+	y8
							842.424191	1+	y7
							729.340127	1+	y6
		APAAEPAIGEHER	light	674.336215	2+	23.1	1037.501048	1+	y9
							908.458455	1+	y8
							627.284514	1+	y5
			heavy	679.340349	2+	23.1	1047.509317	1+	y9
							918.466724	1+	y8
							637.292783	1+	y5
		AAPAAC[+57.0]FAGPPAAPAK	light	734.374286	2+	24.9	779.441014	1+	y9
							708.403901	1+	y8
							651.382437	1+	y7
			heavy	738.381385	2+	24.9	787.455213	1+	y9
							716.4181	1+	y8
							659.396636	1+	y7
CEBPZ	P53569	MLSAILTGVNR	light	587.834265	2+	20.5	930.536706	1+	y9
							659.383499	1+	y6
							546.299435	1+	y5
			heavy	592.8384	2+	20.5	940.544975	1+	y9
							669.391768	1+	y6
							556.307704	1+	y5
		ELLITDLLPDSR	light	692.887749	2+	23.7	916.473437	1+	y8
							587.314751	1+	y5
							474.230687	1+	y4
			heavy	697.891883	2+	23.7	926.481706	1+	y8
							597.32302	1+	y5
							484.238956	1+	y4
CREB1	Q01147	ILNDLSSDAPGVPR	light	727.385905	2+	24.7	885.442471	1+	y9
							798.410442	1+	y8
							525.314357	1+	y5
			heavy	732.390039	2+	24.7	895.45074	1+	y9
							808.418711	1+	y8
							535.322626	1+	y5
			light	531.290548	2+	18.8	849.421342	1+	y8
							650.32565	1+	y6

CSTF3	Q99LI7	LVAQFPSSGR					503.257236	1+	y5
CSTF3	Q99LI7	LVAQFPSSGR	heavy	536.294682	2+	18.8	859.429611	1+	y8
CSTF3	Q99LI7	LVAQFPSSGR	heavy	536.294682	2+	18.8	660.333919	1+	y6
CSTF3	Q99LI7	LVAQFPSSGR	heavy	536.294682	2+	18.8	513.265505	1+	y5
CTBP1	O88712	IGSGFDNIDIK	light	589.806228	2+	20.6	864.446159	1+	y7
CTBP1	O88712	IGSGFDNIDIK	light	589.806228	2+	20.6	717.377745	1+	y6
CTBP1	O88712	IGSGFDNIDIK	light	589.806228	2+	20.6	602.350802	1+	y5
CTBP1	O88712	IGSGFDNIDIK	heavy	593.813327	2+	20.6	872.460358	1+	y7
CTBP1	O88712	IGSGFDNIDIK	heavy	593.813327	2+	20.6	725.391944	1+	y6
CTBP1	O88712	IGSGFDNIDIK	heavy	593.813327	2+	20.6	610.365001	1+	y5
CTBP1	O88712	QGAFLVNTAR	light	538.795997	2+	19.1	673.399149	1+	y6
CTBP1	O88712	QGAFLVNTAR	light	538.795997	2+	19.1	560.315085	1+	y5
CTBP1	O88712	QGAFLVNTAR	light	538.795997	2+	19.1	461.246672	1+	y4
CTBP1	O88712	QGAFLVNTAR	heavy	543.800132	2+	19.1	683.407418	1+	y6
CTBP1	O88712	QGAFLVNTAR	heavy	543.800132	2+	19.1	570.323354	1+	y5
CTBP1	O88712	QGAFLVNTAR	heavy	543.800132	2+	19.1	471.254941	1+	y4
CTBP1	O88712	VGQAVALR	light	407.250694	2+	15.1	714.425699	1+	y7
CTBP1	O88712	VGQAVALR	light	407.250694	2+	15.1	529.345657	1+	y5
CTBP1	O88712	VGQAVALR	light	407.250694	2+	15.1	458.308544	1+	y4
CTBP1	O88712	VGQAVALR	heavy	412.254829	2+	15.1	724.433968	1+	y7
CTBP1	O88712	VGQAVALR	heavy	412.254829	2+	15.1	539.353926	1+	y5
CTBP1	O88712	VGQAVALR	heavy	412.254829	2+	15.1	468.316813	1+	y4
CTNND1	P30999	QDVYGPQPQVR	light	643.828026	2+	22.2	781.431512	1+	y7
CTNND1	P30999	QDVYGPQPQVR	light	643.828026	2+	22.2	724.410049	1+	y6
CTNND1	P30999	QDVYGPQPQVR	light	643.828026	2+	22.2	499.298707	1+	y4
CTNND1	P30999	QDVYGPQPQVR	heavy	648.83216	2+	22.2	791.439781	1+	y7
CTNND1	P30999	QDVYGPQPQVR	heavy	648.83216	2+	22.2	734.418318	1+	y6
CTNND1	P30999	QDVYGPQPQVR	heavy	648.83216	2+	22.2	509.306976	1+	y4
CTNND1	P30999	SDFQVNLNNASR	light	682.831297	2+	23.4	887.469354	1+	y8
CTNND1	P30999	SDFQVNLNNASR	light	682.831297	2+	23.4	788.40094	1+	y7
CTNND1	P30999	SDFQVNLNNASR	light	682.831297	2+	23.4	561.273949	1+	y5
CTNND1	P30999	SDFQVNLNNASR	heavy	687.835431	2+	23.4	897.477623	1+	y8
CTNND1	P30999	SDFQVNLNNASR	heavy	687.835431	2+	23.4	798.409209	1+	y7
CTNND1	P30999	SDFQVNLNNASR	heavy	687.835431	2+	23.4	571.282218	1+	y5
EEF1A1	P10126	IGGIGTVPVGR	light	513.30874	2+	18.3	685.399149	1+	y7
EEF1A1	P10126	IGGIGTVPVGR	light	513.30874	2+	18.3	628.377686	1+	y6
EEF1A1	P10126	IGGIGTVPVGR	light	513.30874	2+	18.3	428.261593	1+	y4
EEF1A1	P10126	IGGIGTVPVGR	heavy	518.312875	2+	18.3	695.407418	1+	y7
EEF1A1	P10126	IGGIGTVPVGR	heavy	518.312875	2+	18.3	638.385955	1+	y6
EEF1A1	P10126	IGGIGTVPVGR	heavy	518.312875	2+	18.3	438.269862	1+	y4
EEF1A1	P10126	YYVTIIDAPGHR	light	702.867151	2+	24	765.400212	1+	y7
EEF1A1	P10126	YYVTIIDAPGHR	light	702.867151	2+	24	652.316148	1+	y6
EEF1A1	P10126	YYVTIIDAPGHR	light	702.867151	2+	24	537.289205	1+	y5
EEF1A1	P10126	YYVTIIDAPGHR	heavy	707.871285	2+	24	775.408481	1+	y7
EEF1A1	P10126	YYVTIIDAPGHR	heavy	707.871285	2+	24	662.324417	1+	y6
EEF1A1	P10126	YYVTIIDAPGHR	heavy	707.871285	2+	24	547.297474	1+	y5
ELAVL1	P70372	DANLYISGLPR	light	609.827494	2+	21.2	805.456664	1+	y7
ELAVL1	P70372	DANLYISGLPR	light	609.827494	2+	21.2	642.393336	1+	y6
ELAVL1	P70372	DANLYISGLPR	light	609.827494	2+	21.2	529.309272	1+	y5
ELAVL1	P70372	DANLYISGLPR	heavy	614.831629	2+	21.2	815.464933	1+	y7
ELAVL1	P70372	DANLYISGLPR	heavy	614.831629	2+	21.2	652.401605	1+	y6
ELAVL1	P70372	DANLYISGLPR	heavy	614.831629	2+	21.2	539.317541	1+	y5
ELAVL1	P70372	SLFSSIGEVESAK	light	677.348457	2+	23.2	919.473102	1+	y9
ELAVL1	P70372	SLFSSIGEVESAK	light	677.348457	2+	23.2	832.441074	1+	y8
ELAVL1	P70372	SLFSSIGEVESAK	light	677.348457	2+	23.2	719.35701	1+	y7
ELAVL1	P70372	SLFSSIGEVESAK	heavy	681.355556	2+	23.2	927.487301	1+	y9
ELAVL1	P70372	SLFSSIGEVESAK	heavy	681.355556	2+	23.2	840.455273	1+	y8
ELAVL1	P70372	SLFSSIGEVESAK	heavy	681.355556	2+	23.2	727.371209	1+	y7
		IFEYETQR	light	543.266738	2+	19.2	972.442137	1+	y7
		IFEYETQR	light	543.266738	2+	19.2	825.373723	1+	y6
		IFEYETQR	light	543.266738	2+	19.2	696.33113	1+	y5
		IFEYETQR					982.450406	1+	y7

EMD	O08579		heavy	548.270873	2+	19.2	835.381992	1+	y6
							706.339399	1+	y5
		TYGEPESVGMSK	light	642.79246	2+	22.2	834.40258	1+	y8
							608.307223	1+	y6
							422.206781	1+	y4
			heavy	646.799559	2+	22.2	842.416779	1+	y8
							616.321422	1+	y6
							430.22098	1+	y4
FABP4	P04117	EVGVGFATR	light	468.250891	2+	17	707.383499	1+	y7
							551.293622	1+	y5
							494.272158	1+	y4
			heavy	473.255026	2+	17	717.391768	1+	y7
							561.301891	1+	y5
							504.280427	1+	y4
		LVSSENFDDYMK	light	724.324124	2+	24.6	932.381845	1+	y7
							818.338917	1+	y6
							671.270503	1+	y5
			heavy	728.331224	2+	24.6	940.396044	1+	y7
							826.353116	1+	y6
							679.284702	1+	y5
FLNA	Q8BTM8	SPFEVYVDK	light	542.271489	2+	19.2	752.382496	1+	y6
							623.339903	1+	y5
							524.271489	1+	y4
			heavy	546.278589	2+	19.2	760.396695	1+	y6
							631.354102	1+	y5
							532.285688	1+	y4
		YNDQHIPGSPFTAR	light	801.886603	2+	27	832.431178	1+	y8
							735.378414	1+	y7
							591.324922	1+	y5
			heavy	806.890738	2+	27	842.439447	1+	y8
							745.386683	1+	y7
							601.333191	1+	y5
FOXK1	P42128	TPFGPLSSR	light	481.258716	2+	17.3	763.409714	1+	y7
							616.3413	1+	y6
							559.319837	1+	y5
			heavy	486.262851	2+	17.3	773.417983	1+	y7
							626.349569	1+	y6
							569.328106	1+	y5
GSK3B	Q9WV60	LLEYTPTAR	light	532.292756	2+	18.9	837.410108	1+	y7
							708.367515	1+	y6
							444.256508	1+	y4
			heavy	537.296891	2+	18.9	847.418377	1+	y7
							718.375784	1+	y6
							454.264777	1+	y4
		TPPEAIALC[+57.0]SR	light	607.81353	2+	21.1	790.423984	1+	y7
							719.386871	1+	y6
							606.302807	1+	y5
			heavy	612.817664	2+	21.1	800.432253	1+	y7
							729.39514	1+	y6
							616.311076	1+	y5
HNRNPA2B1	O88569	GFGFVTFDDHDPVDK	light	565.926493	3+	23.8	825.373723	1+	y7
							458.260925	1+	y4
			heavy	568.597893	3+	23.8	833.387922	1+	y7
							466.275124	1+	y4
		GGNFGFGDSR	light	507.225405	2+	18.1	638.289265	1+	y6
							581.267801	1+	y5
			heavy	512.229539	2+	18.1	648.297534	1+	y6
							591.27607	1+	y5
		GGGGNFGPGPGSNFR	light	689.318356	2+	23.6	888.432241	1+	y9
							831.410777	1+	y8
			heavy	694.322491	2+	23.6	898.44051	1+	y9
							841.419046	1+	y8

HNRNPAB	Q99020	IFVGGLNPEATEEK	light	752.388113	2+	25.5	917.421067	1+	y8
							803.378139	1+	y7
			heavy	756.395213	2+	25.5	925.435266	1+	y8
							811.392338	1+	y7
		EVYQQQQYGSGGR	light	750.347311	2+	25.4	980.454433	1+	y9
							852.395855	1+	y8
			heavy	755.351445	2+	25.4	990.462702	1+	y9
							862.404124	1+	y8
LMNA	P48678	AAYEAELGDAR	light	583.277834	2+	20.4	731.368243	1+	y7
							660.33113	1+	y6
							418.204472	1+	y4
			heavy	588.281969	2+	20.4	741.376512	1+	y7
							670.339399	1+	y6
							428.212741	1+	y4
		ITESEEVVSR	light	574.793317	2+	20.1	718.372994	1+	y6
							589.330401	1+	y5
							460.287808	1+	y4
			heavy	579.797452	2+	20.1	728.381263	1+	y6
							599.33867	1+	y5
							470.296077	1+	y4
		EGDLLAAQAR	light	522.277637	2+	18.6	629.372935	1+	y6
							516.288871	1+	y5
							445.251757	1+	y4
			heavy	527.281772	2+	18.6	639.381204	1+	y6
							526.29714	1+	y5
							455.260026	1+	y4
MGMT	P26187	IAAEIAQAEEQAR	light	700.36243	2+	23.9	831.395521	1+	y7
							703.336943	1+	y6
							632.299829	1+	y5
			heavy	705.366564	2+	23.9	841.40379	1+	y7
							713.345212	1+	y6
							642.308098	1+	y5
		FGETVSYQQLAALAGNPK	light	947.48889	2+	31.3	670.38825	1+	y7
							486.267073	1+	y5
							415.229959	1+	y4
			heavy	951.495989	2+	31.3	678.402449	1+	y7
							494.281272	1+	y5
							423.244158	1+	y4
MYBBP1A	Q7TPV4	TLIDDDNPPVSFVK	light	780.40122	2+	26.3	887.498529	1+	y8
							773.455602	1+	y7
							480.28166	1+	y4
			heavy	784.40832	2+	26.3	895.512728	1+	y8
							781.469801	1+	y7
							488.295859	1+	y4
		QHFSFPLDDR	light	631.301644	2+	21.8	996.478522	1+	y8
							849.410108	1+	y7
							615.309666	1+	y5
			heavy	636.305778	2+	21.8	1006.486791	1+	y8
							859.418377	1+	y7
							625.317935	1+	y5
		NAASQQDAVTEGAMPAATGK	light	959.452178	2+	31.7	1132.566685	1+	y12
							675.349422	1+	y7
							544.308937	1+	y6
			heavy	963.459278	2+	31.7	1140.580884	1+	y12
							683.363621	1+	y7
							552.323136	1+	y6
		LSQVNGATPVSPIEPESK	light	926.98618	2+	30.7	1082.572816	1+	y10
							799.41961	1+	y7
							460.240189	1+	y4
			heavy	930.993279	2+	30.7	1090.587015	1+	y10
							807.433809	1+	y7
							468.254388	1+	y4

NEDD4	P46935	LQNVAITGPAVPYSR	light	793.438472	2+	26.7	947.494506	1+	y9
							846.446828	1+	y8
							522.267073	1+	y4
			heavy	798.442606	2+	26.7	957.502776	1+	y9
							856.455097	1+	y8
							532.275342	1+	y4
		TGGSEIVVTNK	light	552.798403	2+	19.5	802.466895	1+	y7
							673.424302	1+	y6
							560.340238	1+	y5
			heavy	556.805502	2+	19.5	810.481094	1+	y7
							681.438501	1+	y6
							568.354437	1+	y5
NFKB1	P25799	VFETLEAR	light	482.758549	2+	17.4	865.441408	1+	y7
							718.372994	1+	y6
							589.330401	1+	y5
			heavy	487.762684	2+	17.4	875.449677	1+	y7
							728.381263	1+	y6
							599.33867	1+	y5
NONO	Q99K48	FAC[+57.0]HSASLTVR	light	624.811322	2+	21.6	870.479191	1+	y8
							733.420279	1+	y7
							646.38825	1+	y6
			heavy	629.815456	2+	21.6	880.48746	1+	y8
							743.428548	1+	y7
							656.396519	1+	y6
NSUN2	Q1HFZ0	IITVSMEDVK	light	567.807382	2+	19.9	708.323267	1+	y6
							621.291239	1+	y5
							490.250754	1+	y4
			heavy	571.814481	2+	19.9	716.337466	1+	y6
							629.305438	1+	y5
							498.264953	1+	y4
		LFEHYYQELK	light	685.342977	2+	23.5	843.424696	1+	y6
							680.361367	1+	y5
							517.298038	1+	y4
			heavy	689.350077	2+	23.5	851.438895	1+	y6
							688.375566	1+	y5
							525.312237	1+	y4
		TLLTQENPFFR	light	683.361701	2+	23.4	809.394064	1+	y6
							680.351471	1+	y5
							566.308544	1+	y4
			heavy	688.365836	2+	23.4	819.402333	1+	y6
							690.35974	1+	y5
							576.316813	1+	y4
PPARG	P37238	LLAEISSDIDQLNPESADLR	light	1100.060605	2+	35.9	901.437386	1+	y8
							787.394458	1+	y7
							561.299101	1+	y5
			heavy	1105.064739	2+	35.9	911.445655	1+	y8
							797.402727	1+	y7
							571.30737	1+	y5
		SVEAVQEITEYAK	light	733.872296	2+	24.9	1080.557166	1+	y9
							853.430175	1+	y7
							611.303518	1+	y5
			heavy	737.879395	2+	24.9	1088.571365	1+	y9
							861.444374	1+	y7
							619.317717	1+	y5
		ILELDQELTK	light	601.337361	2+	20.9	733.37266	1+	y6
							618.345717	1+	y5
							490.287139	1+	y4
			heavy	605.34446	2+	20.9	741.386859	1+	y6
							626.359916	1+	y5
							498.301338	1+	y4
			light	810.938836	2+	27.2	895.499592	1+	y7
							638.398421	1+	y5

RAD50	P70388	LIQDQQEQIQHLK					525.314357	1+	y4
			heavy	814.945935	2+	27.2	903.513791	1+	y7
							646.41262	1+	y5
							533.328556	1+	y4
		SELQQLEGSSDR	light	674.820595	2+	23.1	763.358073	1+	y7
							650.274009	1+	y6
							521.231415	1+	y5
			heavy	679.824729	2+	23.1	773.366342	1+	y7
							660.282278	1+	y6
							531.239684	1+	y5
		VC[+57.0]LTDVTIMER	light	668.833605	2+	23	863.429129	1+	y7
							748.402186	1+	y6
							649.333772	1+	y5
			heavy	673.83774	2+	23	873.437398	1+	y7
							758.410455	1+	y6
							659.342041	1+	y5
RCOR1	Q8CFE3	VGPQYQAAVPDFDPAK	light	851.925394	2+	28.5	1030.520387	1+	y10
							888.446159	1+	y8
							789.377745	1+	y7
			heavy	855.932494	2+	28.5	1038.534586	1+	y10
							896.460358	1+	y8
							797.391944	1+	y7
RUNX2	Q08775	GTGLPAITDVPR	light	598.835319	2+	20.9	868.488693	1+	y8
							700.398815	1+	y6
							587.314751	1+	y5
			heavy	603.839454	2+	20.9	878.496962	1+	y8
							710.407084	1+	y6
							597.32302	1+	y5
RUVBL1	P60122	AVLLAGPPGTGK	light	540.824223	2+	19.1	684.367515	1+	y8
							613.330401	1+	y7
							556.308937	1+	y6
			heavy	544.831323	2+	19.1	692.381714	1+	y8
							621.3446	1+	y7
							564.323136	1+	y6
		EAC[+57.0]GVIVELIK	light	615.841756	2+	21.4	714.476003	1+	y6
							601.391939	1+	y5
							502.323525	1+	y4
			heavy	619.848855	2+	21.4	722.490202	1+	y6
							609.406138	1+	y5
							510.337724	1+	y4
		QAASGLVGQENAR	light	650.833839	2+	22.4	943.495569	1+	y9
							773.390041	1+	y7
							674.321627	1+	y6
			heavy	655.837974	2+	22.4	953.503838	1+	y9
							783.39831	1+	y7
							684.329896	1+	y6
		TALALAIAQELGSK	light	693.403566	2+	23.7	916.509822	1+	y9
							845.472708	1+	y8
							732.388644	1+	y7
			heavy	697.410666	2+	23.7	924.524021	1+	y9
							853.486907	1+	y8
							740.402843	1+	y7
		VPFC[+57.0]PMVGSEVYSTEIK	light	971.968087	2+	32.1	1439.708658	1+	y13
							1112.546996	1+	y10
							577.319168	1+	y5
			heavy	975.975187	2+	32.1	1447.722857	1+	y13
							1120.561195	1+	y10
							585.333367	1+	y5
		LLIVSTSPYSEK	light	668.871568	2+	23	898.415253	1+	y8
							811.383225	1+	y7
							623.303518	1+	y5
							906.429452	1+	y8

			heavy	672.878667	2+	23	819.397424	1+	y7
							631.317717	1+	y5
RUVBL2	Q9WTM5	TQGFLALFSGDTGEIK	light	842.433052	2+	28.2	1137.57863	1+	y11
							953.457452	1+	y9
							806.389038	1+	y8
			heavy	846.440151	2+	28.2	1145.592829	1+	y11
							961.471651	1+	y9
							814.403237	1+	y8
		VYSLFLDESR	light	614.814053	2+	21.3	766.372994	1+	y6
							619.30458	1+	y5
							506.220516	1+	y4
			heavy	619.818187	2+	21.3	776.381263	1+	y6
							629.312849	1+	y5
							516.228785	1+	y4
SMARCA5	Q91ZW3	TEQEEDEELLTESSK	light	883.894351	2+	29.4	1150.54739	1+	y10
							906.477853	1+	y8
							777.43526	1+	y7
			heavy	887.90145	2+	29.4	1158.561589	1+	y10
							914.492052	1+	y8
							785.449459	1+	y7
		ESEITDEDIDGILER	light	867.407428	2+	28.9	702.37808	1+	y6
							587.351137	1+	y5
							417.245609	1+	y3
			heavy	872.411563	2+	28.9	712.386349	1+	y6
							597.359406	1+	y5
							427.253878	1+	y3
SMARCC1	P97496	TLAGLVVQLLQFQEDAFGK	light	693.049123	3+	28.6	941.436323	1+	y8
							794.367909	1+	y7
							537.266738	1+	y5
			heavy	695.720523	3+	28.6	949.450522	1+	y8
							802.382108	1+	y7
							545.280937	1+	y5
SMARCC2	Q6PDG5	AGGSLC[+57.0]HILAAAYK	light	716.374286	2+	24.4	1046.545162	1+	y9
							886.514514	1+	y8
							636.371538	1+	y6
							523.287474	1+	y5
			heavy	720.381385	2+	24.4	1054.559361	1+	y9
							894.528713	1+	y8
							644.385737	1+	y6
							531.301673	1+	y5
		YIQAEPPTNK	light	580.800945	2+	20.3	685.351531	1+	y6
							556.308937	1+	y5
							459.256174	1+	y4
			heavy	584.808045	2+	20.3	693.36573	1+	y6
							564.323136	1+	y5
							467.270373	1+	y4
SRSF1	Q6PDM2	EAGDVC[+57.0]YADVYR	light	709.306266	2+	24.2	946.408728	1+	y7
							786.37808	1+	y6
							623.314751	1+	y5
			heavy	714.3104	2+	24.2	956.416997	1+	y7
							796.386349	1+	y6
							633.32302	1+	y5
		DGTGVVEFVR	light	539.780013	2+	19.1	805.456664	1+	y7
							649.366787	1+	y5
							550.298373	1+	y4
			heavy	544.784147	2+	19.1	815.464933	1+	y7
							659.375056	1+	y5
							560.306642	1+	y4
		EDMTYAVR	light	492.726392	2+	17.7	740.375971	1+	y6
							609.335487	1+	y5
							508.287808	1+	y4
							750.38424	1+	y6

			heavy	497.730526	2+	17.7	619.343756	1+	y5
							518.296077	1+	y4
TRIM28	Q62318	LDLDLTSDSQPPVFK	light	837.932885	2+	28	802.445765	1+	y7
							587.355159	1+	y5
							490.302396	1+	y4
			heavy	841.939984	2+	28	810.459964	1+	y7
							595.369358	1+	y5
							498.316595	1+	y4
		SGEGEVSGLLR	light	552.288202	2+	19.5	644.408986	1+	y6
							545.340572	1+	y5
							458.308544	1+	y4
			heavy	557.292337	2+	19.5	654.417255	1+	y6
							555.348841	1+	y5
							468.316813	1+	y4
		DHQYQFLEDAVR	light	760.860054	2+	25.7	977.505071	1+	y8
							849.446494	1+	y7
							702.37808	1+	y6
			heavy	765.864189	2+	25.7	987.51334	1+	y8
							859.454763	1+	y7
							712.386349	1+	y6
UBAP2L	Q80X50	LDFIGVEGSNYPR	light	733.867348	2+	24.9	978.463935	1+	y9
							822.374057	1+	y7
							693.331464	1+	y6
			heavy	738.871482	2+	24.9	988.472204	1+	y9
							832.382326	1+	y7
							703.339733	1+	y6
		DGSLASNPYSGDLTK	light	762.862459	2+	25.8	994.484001	1+	y9
							880.441074	1+	y8
							620.324982	1+	y6
			heavy	766.869559	2+	25.8	1002.4982	1+	y9
							888.455273	1+	y8
							628.339181	1+	y6
WDR5	P61965	YILAATLDNTLK	light	668.37956	2+	23	875.483273	1+	y8
							804.446159	1+	y7
							703.398481	1+	y6
			heavy	672.386659	2+	23	883.497472	1+	y8
							812.460358	1+	y7
							711.41268	1+	y6
ZNF326	O88291	ESVLTATSILNNPIVK	light	849.985452	2+	28.4	797.487965	1+	y7
							684.403901	1+	y6
							456.318046	1+	y4
			heavy	853.992551	2+	28.4	805.502164	1+	y7
							692.4181	1+	y6
							464.332245	1+	y4
		NQGGSSWEAPYSR	light	719.820929	2+	24.5	722.34678	1+	y6
							593.304186	1+	y5
							522.267073	1+	y4
			heavy	724.825063	2+	24.5	732.355049	1+	y6
							603.312455	1+	y5
							532.275342	1+	y4
