# Supplementary material for: Using SRM-MS to quantify nuclear protein abundance differences between adipose tissue depots of insulin-resistant mice
Source: J Lipid Res. 2015 May;56(5):1068–78. doi: 10.1194/jlr.D056317 (PMC4409283; doi:10.1194/jlr.D056317)
Supplement: Supplemental Data [file supp_D056317_jlr.D056317-7.pdf]

Supplementary Table S2: List of primers used to make siRNA

Gene Name		Sequence
Cand1	GSP	CGTAAGAAGAGTGGCCTTGG
		TGGAGCTGATCTGTGACTGG
	T7	gcgtaatacgactcactataggATTCTGCGGCCCATAACA
		gcgtaatacgactcactataggAGAGGGCTCTTCTCTGCCTC
Cebpa	GSP	cccacttgcagttccagatc
		gaaaccatcctctgggtctc
	T7	gcgtaatacgactcactataggagaccaccatgcacctac
		gcgtaatacgactcactataggcaaggcggtcccgcag
Cebpb	GSP	ACGACTTCCTCTCCGACCTC
		CAGCTGCTCCACCTTCTTCT
	T7	gcgtaatacgactcactataggCAAGCCGAGCAAGAAGCC
		gcgtaatacgactcactataggCTTCTGCAGCCGCTCGTT
Cstf3	GSP	gcggaatatgtcccagagaag
		taaacctgtgcctctcgaatg
	T7	gcgtaatacgactcactataggaaatccatatgaccttgatgc
		gcgtaatacgactcactataggtgctccaagcatcaaggtc
Ctnnd1	GSP	AGACGCCTGCCATCTTAGAA
		GGGGGAGAGTGCTATCATCA
	T7	gcgtaatacgactcactataggGCAGATACATCCGCTCTGCT
		gcgtaatacgactcactataggATATGAATGGCTGCTCTGGC
Fabp4	GSP	gtgtgatgcctttgtgggaac
		aaactcttgtggaagtcacgc
	T7	gcgtaatacgactcactataggtggaagcttgtctccagtg
		gcgtaatacgactcactataggaacacattccaccaccagc
Flna	GSP	tgtgaacacaagcaatgcagg
		agggtaccataatgcggtatg
	T7	gcgtaatacgactcactatagggccctttcggttaccattg
		gcgtaatacgactcactataggtgcctgggatatgctcatc
Hnrnpa2b1	GSP	aggaagatactgaggaacacc
		tatctgctccttccaccatag
	T7	gcgtaatacgactcactataggaccttagagattactttgaag
		gcgtaatacgactcactataggacttcctccaggaccatag
Mgmt	GSP	tcggagaaacggtttcttacc
		aatatagtggtgccaccacac
	T7	gcgtaatacgactcactataggagcaattagcagccctgg
		gcgtaatacgactcactataggttccgacacgcgttacatc
Mybbp1a	GSP	gccaaggatattcctagtgac
		ctggccactctccttttcttg
	T7	gcgtaatacgactcactataggcaaagcggaagaaaaaggg
		gcgtaatacgactcactataggaccccactctgtaacagg
Nedd4	GSP	tttggacagacacatcagcac
		ctaatcaacgccatcaaagcc

	T7	gcgtaatacgactcactataggctgaaaaccggaggatcag
		gcgtaatacgactcactataggctgtgtgttctcaattgcc
Nfkb1	GSP	ATGACCTGGACGACTCTTGG
		GATAGCAGTGGGCTGTCTCC
	T7	gcgtaatacgactcactataggTGGAGAAGATGAGGGAGTGG
		gcgtaatacgactcactataggCTGAGTTTGCGGAAGGATGT
Pparg	GSP	tccgaatttttcaagggtgcc
		caagtccttgtagatctcctg
	T7	gcgtaatacgactcactatagggtttcgatccgtagaagcc
		gcgtaatacgactcactatagggtgaaggctcatgtctgtc
Zfp326	GSP	GCAACAGACACTTAATCACCCA
		CTCGCACTGCTCAGTAGCAC
	T7	gcgtaatacgactcactataggTGGAAGGTGTTACTGCAGATG
		gcgtaatacgactcactataggTCCTCTTCCTCCTCCTCCTC
