# Supplementary figures and images for: Using SRM-MS to quantify nuclear protein abundance differences between adipose tissue depots of insulin-resistant mice
Source: J Lipid Res. 2015 May;56(5):1068–78. doi: 10.1194/jlr.D056317 (PMC4409283; doi:10.1194/jlr.D056317)

**Supplementary Figure S6: Relative contributions of each protein to Principal Components #1 and #2.**

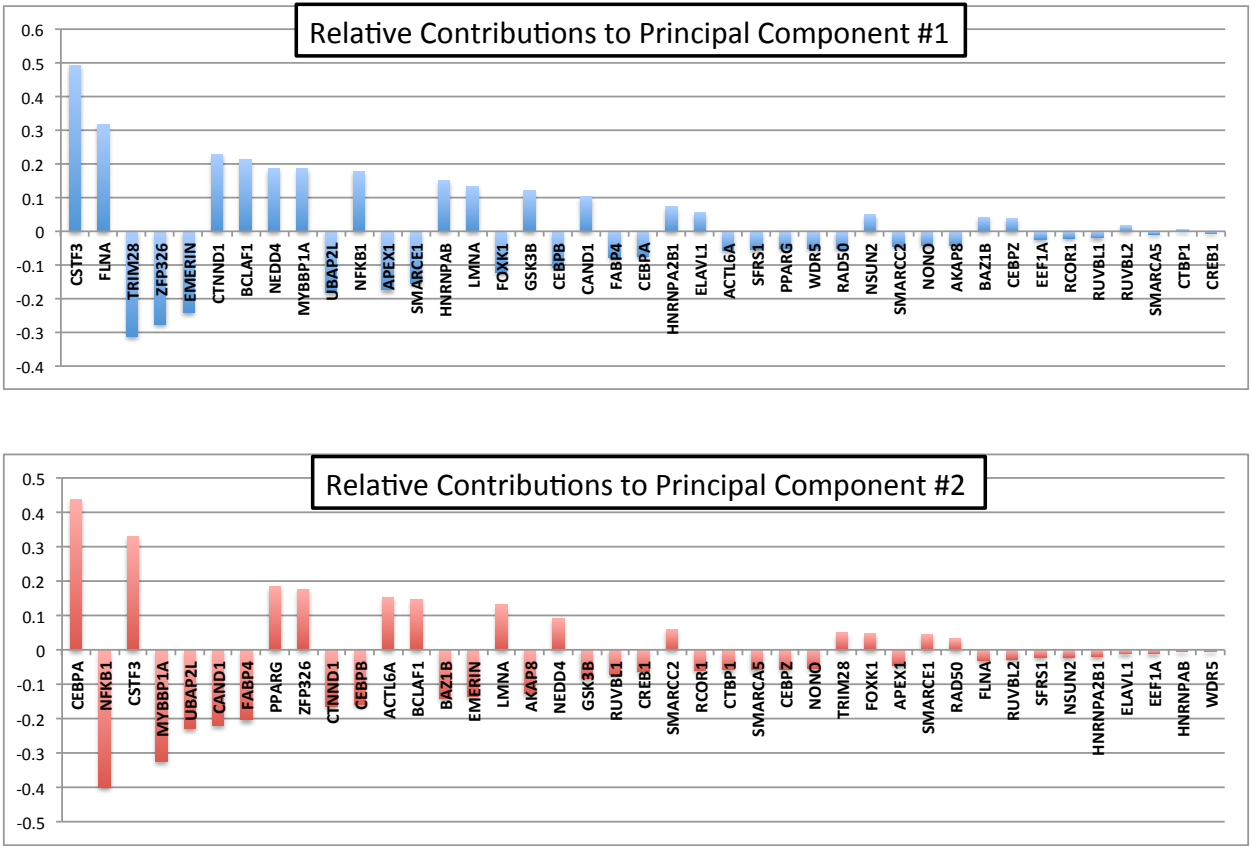

Supplement: Supplemental Data [file supp_D056317_jlr.D056317-9.pdf]
